# A consensus definition and rating scale for minimalist shoes

**DOI:** 10.1186/s13047-015-0094-5

**Published:** 2015-08-19

**Authors:** Jean-Francois Esculier, Blaise Dubois, Clermont E. Dionne, Jean Leblond, Jean-Sébastien Roy

**Affiliations:** Faculty of Medicine, Laval University, Quebec City, QC Canada; Centre for Interdisciplinary Research in Rehabilitation and Social Integration, Quebec Rehabilitation Institute, 525, Boulevard Wilfrid Hamel, Quebec City, QC Canada, G1M 2S8; The Running Clinic, Quebec City, QC Canada; Axe Santé des populations et pratiques optimales en santé, Centre de recherche FRQS du CHU de Québec, Quebec City, QC Canada

**Keywords:** Running, Consensus statement, Running injuries, Foot, Delphi, Validity, Reliability, Footwear

## Abstract

**Background:**

While minimalist running shoes may have an influence on running biomechanics and on the incidence of overuse injuries, the term "minimalist" is currently used without standardisation. The objectives of this study were to reach a consensus on a standard definition of minimalist running shoes, and to develop and validate a rating scale that could be used to determine the degree of minimalism of running shoes, the Minimalist Index (MI).

**Methods:**

For this modified Delphi study, 42 experts from 11 countries completed four electronic questionnaires on an optimal definition of minimalist shoes and on elements to include within the MI. Once MI was developed following consensus, 85 participants subjectively ranked randomly assigned footwear models from the most to the least minimalist and rated their degree of minimalism using visual analog scales (VAS), before evaluating the same footwear models using MI. A subsample of thirty participants reassessed the same shoes on another occasion. Construct validity and inter- and intra-rater reliability (intraclass correlation coefficients [ICC]; Gwet's AC1) of MI were evaluated.

**Results:**

The following definition of minimalist shoes was agreed upon by 95 % of participants: "Footwear providing minimal interference with the natural movement of the foot due to its high flexibility, low heel to toe drop, weight and stack height, and the absence of motion control and stability devices". Characteristics to be included in MI were weight, flexibility, heel to toe drop, stack height and motion control/stability devices, each subscale carrying equal weighing (20 %) on final score. Total MI score was highly correlated with VAS (*r* = 0.91). A significant rank effect (p < 0.001) confirmed the MI's discriminative validity. Excellent intra- and inter-rater reliability was found for total MI score (ICC = 0.84-0.99) and for weight, stack height, heel to toe drop and flexibility subscales (AC1 = 0.82-0.99), while good inter-rater reliability was found for technologies (AC1 = 0.73).

**Conclusion:**

This standardised definition of minimalist shoes developed by an international panel of experts will improve future research on minimalist shoes and clinical recommendations. MI's adequate validity and reliability will allow distinguishing running shoes based on their degree of minimalism, and may help to decrease injuries related to footwear transition.

**Electronic supplementary material:**

The online version of this article (doi:10.1186/s13047-015-0094-5) contains supplementary material, which is available to authorized users.

## Background

Running is a sport that has gained popularity in recent years [1], but between 19.4 and 79.3 % of runners sustain a running-related injury in any given year [2]. To address this issue, the running shoe industry developed countless features, such as increased cushioning, elevated heel, as well as motion control and stability technologies, aiming at protecting the body from the mechanical stress caused by running. It is also commonly believed that runners with different foot types (*pes planus*, normal foot, *pes cavus*) or mechanics (pronating foot, neutral foot, supinating foot) should be fitted in different shoes in order to optimise protection. However, the variety of implemented technologies and prescription strategies based on foot type and mechanics have failed to decrease the rate of running-related injuries [3–7]. Furthermore, some studies have suggested that cushioning properties in modern technologic shoes may alter natural running biomechanics [8] by modifying kinematics [9–14], kinetics [13–16] and muscle activation patterns [17, 18]. It has also been reported that additional distal weight caused by heavier traditional shoes during running may lead to higher oxygen consumption and energy expenditure, therefore being detrimental to running economy [19–22].

As a reaction to these findings, thinner and less structured shoes were gradually brought back on the market. The aim of these "minimalist shoes" was to promote barefoot-like biomechanics by interfering in a lesser extent with the natural movement of the foot [23]. However, inconsistent findings have been reported in the literature regarding the effects of minimalist shoes on running biomechanics. While some have suggested that minimalist shoes promote better impact-moderating behaviour similar to barefoot running [13, 14, 16, 24–27], others have found no such effect [28–31]. Discordance can also be found between the small number of studies that investigated the effects of minimalist shoes on the incidence of running injuries [32–34]. Such disparities may be related to the different shoe models that have been utilised, some of which are closer to barefoot than others. Specific characteristics, either alone or in combination, are commonly but not uniformly utilised when it comes to describing minimalist shoes in order to study their potential effects: low stack height (thickness of shoe at the heel, including outsole, midsole and insole), low heel to toe drop (difference in thickness between heel and forefoot), low weight, high flexibility, anatomical last (wide toe-box), and very few motion control and stability technologies [23, 33]. Thus, the term "minimalist" is currently used in the literature and on the shoe market without standardisation. Furthermore, no rating scale is available to state potential differences between different types of running shoes. Terms such as "partial minimalist" and "full minimalist" have been reported in the literature, although no further details were provided on how these words were selected [33]. Such lack of consensus is responsible for confusion when trying to determine the effects of minimalist shoes, and explains difficulties when performing comparisons between studies. Therefore, through a modified Delphi design, the first objective of this study was to reach a consensus on a standard definition of minimalist shoes, and to develop a rating scale that could be used to determine the degree of minimalism of running shoes, the Minimalist Index (MI). Thereafter, the second objective was to validate and evaluate the psychometric properties (construct and discriminative validity, intra- and inter-rater reliability) of the newly developed MI.

## Methods

### Delphi study

**Study design.** A modified Delphi approach was used to address the first objective of this study [35]. Participants had to complete four rounds of questions using electronic questionnaires (*SurveyMonkey.com*) relating to the optimal definition of minimalist shoes and to the development of a measuring scale, the MI. Consensus was defined by 67 % agreement of experts [36].

**Participants**. Names of experts in the field of running shoes were retrieved through scientific electronic databases and over the Internet. Additional experts were contacted based on suggestions from participants during round one. Following email invitation of 62 experts from 13 countries, a heterogeneous panel of 43 experts from 11 countries that primarily included researchers and healthcare practitioners responded favorably to the invitation (participation: 69.3 %). Although special attention was taken to obtain large geographical coverage, 58 % of panel experts were located in North America, given the higher positive response rate from the experts from this continent. Formal written consent was not deemed necessary by the local ethics committee. Thus, an informal consent statement was confirmed from each round of questioning.

#### Data collection

*Round 1*. First, fourteen characteristics and claimed effects of minimalist shoes were retrieved following an extensive review of the scientific literature. Participants were asked to rate every item using 11-point Likert scales (0–10), where 0 meant "Not suitable at all for the definition of minimalist shoes", and 10 meant "Should definitely be included within the definition of minimalist shoes". Experts were also invited to submit additional elements that they felt were of importance. Second, participants were asked to give their opinion on items that should be included within a measuring scale aiming to quantify the degree of minimalism of different shoes. They had to rate 10 characteristics, also using 11-point Likert scales, where 0 meant "Not suitable at all for the differentiation of minimalist and traditional shoes" and 10 meant "Should definitely be used for differentiating minimalist from traditional shoes". Once more, participants were invited to suggest additional elements they felt were important in order to quantify the degree of minimalism. Then, all experts were asked to submit their own preferred definition of minimalist shoes and to suggest the names of other experts in the field of running shoes.

*Round 2*. Descriptive statistics of results to Round 1 were established. Items were then classified into "provisionally included" or "provisionally excluded" depending if they reached the *a priori* determined rating threshold of 7/10 for the definition of minimalist shoes, and 8/10 for the MI. Such thresholds were established so that only the most important characteristics were quantified within the rating, while the definition could possibly include additional elements. Panel members were provided with the mean and standard deviation obtained during Round 1 for all items, and were asked whether they agreed to include the items that reached the rating threshold, and to exclude those that did not. Based on the *a priori* agreement threshold, elements that were provisionally classified as included needed 67 % agreement to be included, and elements that were provisionally classified as excluded needed 67 % of disagreement in order to be resubmitted within the next round regarding their inclusion. Experts were also invited to comment their position on each rated element.

*Round 3*. Based on results from the first two rounds, a definition of minimalist shoes was built and included all elements that were agreed upon by the expert panel. Furthermore, a comprehensive review of the literature and running footwear features was performed by the research team, which allowed to build rating scales for characteristics that were judged by the panel as important to include within the MI. The goal of these scales was to differentiate and categorise minimalist and traditional shoes. In Round 3, participants were asked whether they agreed or not with the suggested definition and the use of specific terms, as well as with the suggested 6-point Likert scales that would be part of the MI. In addition, they were asked which proportion of total MI score they thought each characteristic should account for. Participants were encouraged to comment on the definition and on the scales so that they could be improved and resubmitted for agreement in Round 4.

*Round 4*. In this final round, we sought the opinion of the panel on minor changes that were made to rating scales, and on precisions and pictures added to the flexibility assessment. In addition, participants were asked for their permission to be acknowledged within this article. It was mentioned to experts who did not agree with the consensus definition that their opinion would be stated in the article, and that their contribution did not represent endorsement of minimalist shoes.

### Psychometric properties of the MI

**Participants**. A convenience sample of 85 participants was recruited for the validation phase of the MI (including 30 participants who took part in the intra-rater assessment of MI). To be included, participants had to (1) be a researcher (health or sport sciences), a physician, allied health professional or a specialised running shoe retailer/manufacturer, (2) have at least one year of experience in recommending running shoes to injured or uninjured runners, and (3) be aged 18 or higher. Advertisements were made through email blasts and during scientific conferences related to the topics of running and sports (33rd Congress of the International Federation of Sports Medicine; 1st Calgary International Running Symposium). With less than a 20 % error of estimation, such a sample could effectively detect inter-rater agreement above 54 % and intra-rater agreement above 92 % [37]. This study was approved by Laval University Research Ethics Committee, and all subjects signed a detailed consent form prior to participation.

**Data collection**. All participants took part in one testing session of approximately 30 minutes during which validity and inter-rater reliability of the MI was assessed. Ten brand new and unworn running shoe models (left and right; US men's size 9) were selected by the research team to cover the whole spectrum from minimalist to maximalist shoes. All 20 shoes were randomised into 4 different boxes so that duplicates were avoided. Using a random number generator (*randomizer.org*) prior to recruitment, participants were randomly assigned to a box number, which was written in sealed opaque envelopes.

After opening the envelope assigned to their recruitment number, participants were presented with the consensus definition of minimalist shoes. Then, they were asked to rank the assigned shoes from the most minimalist to the least minimalist (1–5, where 1 = least minimalist and 5 = most minimalist) according to their own perception. Participants also had to subjectively rate the degree of minimalism of each shoe using a visual analog scale (0–100, where 100 = extremely minimalist and 0 = not minimalist at all). Following completion of all subjective ratings, the forms were secured into an envelope so that no further changes could be made and no consultation of the forms was possible thereafter. Then, testing using the MI was performed (see Additional file 1, Minimalist Index rating scale). A complete written scoring guide was provided to participants that described precisely how to rate the five MI subscales (see Additional file 2, Minimalist Index instruction guide). Participants who took part to the intra-tester arm of the study were met for a second session at least two days after the initial testing session to rate a second time the same five shoe models with the MI (access to the written scoring guide was still permitted).

**Statistical analyses**. For Delphi rounds 1 and 2, descriptive statistics were calculated (mean and standard deviation) for results to 11-point Likert scales. Agreement rates were expressed in percentage of panel members.

As for determination of MI's psychometric properties, average scores to VAS were calculated for each subjectively attributed ranking (1 to 5). Discriminative validity of the MI was evaluated using a generalised repeated-measure ANOVA [SPSS 22; Generalised Estimating Equations (GEE); distribution = Gamma; link = log; unstructured covariance matrix] by comparing the mean scores on the MI according to their rank. A Pearson correlation was used to evaluate convergent validity between total MI and VAS scores. Inter-rater reliability of MI total score was determined for each group number (1 to 4), and intra-rater reliability was calculated for all participants regardless of assignation using Mixed model absolute intraclass correlation coefficients (ICC [2, 1]). Gwet's AC1 indices with quadratic weighting were used to determine the reliability of the MI's subscales (AgreeStat2013.2, Advanced Analytics, Gaithersburg, MD, USA) [38]. Results were considered significant when *P* < 0.05.

## Results

### Participants

Out of 43 experts who initially accepted to take part in the Delphi study, 42 (97.6 %) completed the first and second rounds, and 39 (90.7 %) completed the third and fourth rounds. The expert panel represented Australia (n = 6), Brazil (n = 1), Canada (n = 8), Denmark (n = 1), France (n = 1), Hong Kong (n = 1), Ireland (n = 1), Luxembourg (n = 1), the Netherlands (n = 1) South Africa (n = 2) and the United States (n = 19). The majority of experts (69 %) had authored peer-reviewed scientific articles on running footwear and/or biomechanics, while the others (31 %) were researchers or clinicians involved in knowledge transfer related to these topics.

As for the reliability study, a total of 50 clinicians including physical therapists (n = 23), physicians (n = 17), podiatrists/pedorthists (n = 6) and chiropractors (n = 4), as well as 27 running shoe retailers/manufacturers representatives and 8 researchers completed the MI (n = 85; 40 women). On average, participants had 9.8 ± 9.2 years of experience in recommending or conducting research on running shoes (range: 1–37 years). The subgroup who performed repeated testing (n = 30; 17 women) had 5.1 ± 5.3 years of experience (range: 1–18).

### Delphi study

**Round 1**. A total of 10 items reached the rating threshold of 7/10 to be provisionally included within the definition of minimalist shoes, and 4 were provisionally excluded (Table 1). Thirty-one additional suggestions were received from the expert panel, which were synthesised to 3 new items being considered for rating during the next round. Regarding the MI, 7 items reached the threshold to be provisionally included, and 3 others did not. One newly suggested item - Presence of outsole lugs - was retained for further consideration (Table 2).

**Table 1:** Mean ratings (SD) (Round 1; range: 0–10) and agreement rates (Round 2; %) for items to be included within the definition

	Round 1	Round 2
	Rating	Inclusion
Provisionally included		
Low heel to toe drop	9.2 (1.8)	88.1
High flexibility	9.1 (1.8)	95.2
Absence of motion control/stability technologies	9.1 (1.9)	97.6
Provide minimal interference to the natural movement of the foot	9.0 (2.6)	88.1
Light weight	8.9 (1.9)	92.9
Low stack height	8.6 (2.3)	88.1
Allow for natural expansion of the forefoot	8.4 (2.6)	85.7
Anatomical last (wide toe-box)	7.3 (3.3)	78.6
Encourage lower limb kinematics similar to barefoot	7.2 (3.8)	61.9
Encourage lower limb kinetics similar to barefoot	7.1 (3.9)	61.9
	Round 1	Round 2
	Rating	Exclusion
Provisionally excluded		
Allow for ground feel	6.9 (3.1)	66.7
Encourage lower limb muscle activation similar to barefoot	6.8 (3.9)	71.4
Absence of sole cushioning^a^	5.6 (3.8)	---
Facilitate afferent feedback similar to barefoot running^a^	4.9 (3.8)	---
Replicate energy expenditure of barefoot running	4.8 (3.5)	83.3
Replicate oxygen consumption of barefoot running	4.6 (3.4)	81.0
Adequate vertical volume of toe-box^a^	3.8 (3.5)	---

^a^Additional items suggested by participants during Round 1. These items did not obtain sufficient ratings to be further considered for inclusion

**Table 2:** Mean ratings (SD) (Round 1; range: 0–10) and agreement rates (Round 2; %) for items to be included within the MI

	Round 1	Round 2
	Rating	Inclusion
Provisionally included		
Heel to toe drop (lower for minimalist)	9.0 (2.1)	92.9
Torsional flexibility (higher for minimalist)	8.8 (1.9)	95.2
Weight (lower for minimalist)	8.8 (1.5)	100
Longitudinal flexibility (higher for minimalist)	8.7 (2.0)	95.2
Stack height (lower for minimalist)	8.6 (2.6)	88.1
Motion control devices (dual/multi-density midsole, rigid heel counter) (fewer for minimalist)	8.5 (2.9)	85.7
Arch support devices (elevated medial insole under foot arch, tensioned medial upper) (fewer for minimalist)	8.5 (2.9)	85.7
	Round 1	Round 2
	Rating	Exclusion
Provisionally excluded		
Upper/cover flexibility (higher for minimalist)	7.8 (2.7)	78.6
Toe-box width (anatomical fit) (higher for minimalist)	7.2 (3.3)	57.1
Sole density (higher for minimalist)	6.5 (3.2)	81.0
Presence of outsole lugs (fewer for minimalist)^a^	2.7 (3.4)	---

^a^Additional item suggested by participants during Round 1. This item did not obtain sufficient rating to be further considered for inclusion

**Round 2**. Eight items reached the agreement threshold for inclusion within the definition of minimalist shoes, while 2 others were eliminated (Table 1). The three new items that were rated by the panel did not get sufficient scores (mean scores < 5.6/10) to be considered within the definition. For the MI, however, the seven provisionally included items reached sufficient agreement to be retained for incorporation within the rating scale (Table 2). It was decided by the research team to combine "motion control technologies" and "stability technologies" within a "motion control and stability technologies" category, as well as "longitudinal flexibility" and "torsional flexibility" within a "flexibility" category with two subscales.

**Round 3**. To optimize the definition's readability, it was agreed on by the panel to include "Allow for natural expansion of the forefoot" and "Anatomical fit" into "Provide minimal interference with the natural movement of the foot". In addition, the expression "heel to toe drop" was preferred over heel to toe "differential", "ramp" or "offset". Therefore, the following definition of minimalist shoes was agreed upon by 95 % of participants: "*Footwear providing minimal interference with the natural movement of the foot due to its high flexibility, low heel to toe drop, weight and stack height, and the absence of motion control and stability devices*". Characteristics to be included in the MI were weight, flexibility, heel to toe drop, stack height and motion control/stability devices. It was determined by the panel that all five items should account for similar weighing within the final score (20 % each). The research team decided that the sum of all subscores should by multiplied by four in order to obtain a maximum total MI score of 100, expressed in percentage. Experts favourably agreed on the 6-point Likert scales that were suggested to rate each characteristic, in which higher scores meant a higher degree of minimalism (Table 3). However, suggestions for modifications were received from 6 participants regarding the stack height scale, and from 8 participants regarding the flexibility scales.

**Table 3:** Agreement of panel members (%) with suggested scales for rating each characteristic

	Round 3	Round 4
Subscales		
Weight	86.8	---
Stack height	81.6	87.2
Heel to toe drop	89.5	---
Flexibility (longitudinal and torsional)	79.0	91.0
Motion control and stability technologies	73.7	---

Weight and heel to toe drop scales were not resubmitted to the panel during Round 4 because of high agreement rates obtained during Round 3. Motion control and stability technologies scale was not resubmitted because no suggestion was received from the panel during Round 3 so that it could be optimised

**Round 4**. As expected, agreement rates for rating scales were increased following suggestions from the experts to shift the "stack height" rating scale to lower values, as well as to improve objective rating procedures for flexibility assessment. Stack height scale agreement increased from 81.6 % to 87.2 %, and flexibility scales increased from 79.0 % to 91.0 % (Table 3; for details on values that were adopted for each scale, see Additional file 1, Minimalist Index rating scale). Out of the 40 panel members who completed at least 3 rounds, one expert did not wish to be acknowledged within this article because he didn't want to be perceived as endorsing minimalist shoes.

### Psychometric properties of the MI

**Validity and reliability**. Descriptive statistics for VAS and MI scores according to rank are presented in Table 4. A rank effect (*p* < 0.001 after a Bonferroni correction) was found, suggesting that MI can accurately differentiate between levels of minimalism. On average, an increase of one rank increases the MI score by 19.3 (R^2^ = 0.98). Scores on the VAS and MI were highly correlated (*r* = 0.91, *p* < 0.001).

**Table 4:** Descriptive data for VAS and MI scores

		Rank 1	Rank 2	Rank 3	Rank 4	Rank 5
VAS	*Mean*	6.4	17.0	50.6	71.7	89.8
	*SD*	9.6	12.8	13.2	11.9	10.5
MI	*Mean*	9.0	17.4	46.4	62.3	82.9
	*SD*	7.5	9.0	9.2	11.6	15.1

VAS, Visual Analog Scale; MI, Minimalist Index; SD, Standard Deviation

Excellent inter-rater reliability was found for total MI score in each group as well as for weight, stack height, heel to toe drop and flexibility subscales (Table 5). Analyses yielded good inter-rater reliability for the technologies subscale (Table 5). As for intra-rater, MI proved to have excellent reliability for total score and for all five subscales (Table 5).

**Table 5:** Inter- and intra-rater reliability indices for MI

	Inter-rater		Intra-rater	
	ICC	95 % C.I.	ICC	95 % C.I.
Total score				
*All groups*	---		0.98	0.97 - 0.98
*Group 1 (n = 24)*	0.84	0.65 - 0.98	---	
*Group 2 (n = 18)*	0.99	0.96 - 0.99	---	
*Group 3 (n = 23)*	0.89	0.73 - 0.99	---	
*Group 4 (n = 20)*	0.94	0.84 - 0.99	---	
	Gwet's AC1	95 % C.I.	Gwet's AC1	95 % C.I.
Weight	0.99	0.97 - 1.00	0.99	0.98 - 1.00
Stack height	0.94	0.89 - 1.00	0.97	0.96 - 0.98
Heel to toe drop	0.82	0.69 - 0.94	0.88	0.84 - 0.92
Technologies	0.73	0.61 - 0.86	0.93	0.91 - 0.96
Flexibility	0.88	0.81 - 0.96	0.93	0.91 - 0.95
*Longitudinal flexibility*	0.86	0.77 - 0.95	0.95	0.93 - 0.97
*Torsional flexibility*	0.87	0.82 - 0.92	0.92	0.90 - 0.95

Group 1: ASICS Gel-Cumulus 15, Inov8 Road-XLite 155, New Balance MR1400TB, New Balance M1080WB3, Nike Free Flyknit 4.0

Group 2: ASICS Gel-Cumulus 15, Inov8 Road-XLite 155, Mizuno Wave Nirvana 7, Saucony Kinvara 5, Vibram Five Fingers Bikila Evo

Group 3: Brooks Glycerin 12, New Balance MR1400TB, New Balance M1080WB3, Saucony Type A6, Vibram Five Fingers Bikila Evo

Group 4: Brooks Glycerin 12, Mizuno Wave Nirvana 7, Nike Free Flyknit 4.0, Saucony Kinvara 5, Saucony Type A6

## Discussion

Despite the recent increase in market shares and the number of investigations on minimalist shoes, our study confirmed the need for a standardised definition of minimalist running shoes. Indeed, when experts initially suggested their own preferred definition of minimalist shoes, we observed many similarities but also several differences. In fact, commonly advocated characteristics of minimalist shoes, such as lightness, high flexibility as well as low stack height and heel to toe drop were observed in the majority of individual definitions and obtained high ratings for inclusion within the standardised definition. However, many experts emphasized that additional research is needed to determine if minimalist shoes really encourage lower limb kinematics, kinetics and muscle activations similar to barefoot running. Studies that have been published so far have reported divergent findings using shoe models that were all classified as minimalist according to their authors' definition; however, characteristics were sometimes very different. This research process confirmed that there was a definite need to develop a rating scale that would allow optimising study designs and comparability between studies to better determine the effects of minimalist shoes.

During Rounds 1 and 2, the panel strongly agreed on the fact that minimalist shoes should not restrict the natural movement of the foot, ideally by having a wide toe-box until the tip of the toes (anatomical fit) that contributes to natural expansion of the forefoot during gait. Since all these elements targeted the same construct, which did not require experimental validation, experts agreed to merge them together within the definition. Participants agreed that minimal interference with the natural movement of the foot could be achieved through high flexibility as well as low weight, stack height, heel to toe drop and the absence of motion control technologies, so that the definition's formulation was acceptable. Even though these characteristics might not consistently influence all runners' biomechanics, at least not according to the current evidence [39–42], they are assumed to minimise the impact on barefoot biomechanics.

Two experts disagreed with the consensus definition; one (RWW) commented that the definition should only include shoe characteristics without mentioning the foot, while the other one (BMN) commented that the definition was too specific and included, in his opinion, characteristics that were not related to minimalist shoes (e.g. flexibility).

Similar to the definition, high ratings were received for weight, stack height, heel to toe drop, flexibility and motion control and stability devices to be included within the MI. As pointed out by experts during the process, it could be argued that a total score is not needed and that all characteristics could be reported to describe a shoe. From a research point of view, a summary of scores to all subscales of the MI is certainly pertinent to explain research findings in more details. However, a total score should be seen as pertinent and needed to inform the running community. A total score in percentage was chosen for its accessibility to the general public, where 100 % represents the highest degree of minimalism, and 0 % represents very maximalist shoes. Thus, the sum of all subscores is multiplied by 4 to obtain the total MI score (see Additional file 1, Minimalist Index rating scale). For example, a runner seeking to buy new shoes can relate on a difference in MI score to guide his transition, and adjust training accordingly so that injury risk is minimised [32, 33]. Although more evidence is needed to establish guidelines, it could be reasonably hypothesised that transitioning from shoes rated 10 % to others rated 30 % within one month is more likely to be safer than switching to shoes rated 80 % within the same timeframe. The name *Minimalist Index* was chosen to reflect higher degrees of minimalism in higher scored shoes, even though it covers the whole spectrum of running footwear. Since the panel determined that all subscores should equally influence the total score, a lighter shoe with higher stack height could be scored the same as a heavier shoe with a lower stack height, for example. Hence, no cut-off value for dichotomising between minimalist or not can be determined at this point. Indeed, comments received about similar weighing between all factors revealed that evidence was lacking to state that specific characteristics should account for a higher proportion of total MI score, therefore accounting for a higher degree of minimalism. To our knowledge, three studies have investigated the effects of different stack heights on running biomechanics [39, 40, 42] and only one study was published regarding the influence of different heel to toe drops [41]. Thus, we suggest that research be conducted to determine if specific characteristics should be perceived as more responsible than others for a decreased interference with the natural movement of the foot, and consequently, for a higher degree of minimalism.

Since the goal of an objective scale like the MI is to quantify the degree of minimalism of a given running shoe regardless of the person wearing it, it would have been suboptimal that items related to fitting (e.g. wide toe-box) influence the MI score given that every runner's foot shape is different. Even if comfort remains the key factor when selecting appropriate running shoes, it seems illogical that improper fitting would be a factor responsible for changing how minimalist is a shoe. Most of the aforementioned characteristics can be easily quantified using a standard man size (United States size 9; United Kingdom size 8; European size 42.5), especially when weight, stack height and heel to toe drop are accurately provided by the manufacturer.

Even though agreement thresholds were reached for all subscales during Round 3, it was decided by the research team to adopt modifications for stack height and flexibility ratings. As expected, agreement rates increased following adjustments, and the detailed assessment of flexibility containing descriptions and pictures of all possible scores was very well received by participants. As for the motion control and stability technologies subscale, it was not modified although it received the lowest agreement rate. In fact, despite a few suggestions for classifying technologies depending on their influence on biomechanics, no alternative was found that was supported by the literature; therefore, it was decided that all devices would account for the same influence within this specific subscore.

It is imperative that rating scales present with adequate psychometric properties before they can be utilised. Based on results from this study, the MI proved to be highly correlated with VAS, to have the ability to differentiate between different degrees of minimalism and to be highly reliable for both inter- and intra-rater assessments [43]. Thus, the MI can be used by retailers and clinicians to provide insightful footwear recommendations to runners.

The rating guide on how to use the MI certainly contributed to high reliability indices obtained in this study. Indeed, detailed instructions provided to participants standardised the rating process so that inter- and intra-rater reliability were excellent. Obviously, weighting the shoe produced very consistent results, except in some shoes for which the weight was close to a cut-off between two possible scores. Similar variations occurred with stack height and heel to toe drop measurements, in addition to a possibility of error secondary to suboptimal use of the electronic caliper. Since heel to toe drop is calculated using two different caliper measurements, it was expected that its reliability be slightly inferior to that of stack height. Of note, means of measurements issued by participants sometimes differed from manufacturer's specifications, potentially because factory measures were calculated without shoe insole. Participants had to assess shoes with the insole, as determined by the experts' consensus on minimalist shoes.

Flexibility and technologies assessment were expected to result in slightly inferior indices of reliability. Despite possible variations in strength applied to determine shoe flexibility, we believe that standardised instructions on the use of only three fingers per hand along with specific descriptions of every rating optimised reliability. As for technologies, it must be noted that inter-rater reliability might have been influenced by variations in materials used by different manufacturers. Hence, different raters may not interpret equally the presence of devices like "rigid heel counter" and "supportive tensioned medial upper". Nonetheless, good reliability was found for this subscale.

This work should be seen as an effort from a group of international experts to improve knowledge in the field of running footwear. Still, this study contains some limitations. First, some continents were less represented in the panel (Asia, Africa, South America), which may have influenced the study results by adding a cultural bias regarding the perception of minimalist shoes. Second, the flexibility as well as motion control and stability devices assessments may contain an evaluator-related bias. The strength applied to the shoe, and the ability to identify technologies within the shoe may vary from one evaluator to the other. To address this issue, a detailed assessment guide was created to standardise evaluation procedures (see Additional file 2, Minimalist Index instruction guide). Third, many more technologies currently exist and others will be developed in the future, but not all of them are included within the MI. However, this panel of experts helped determine the most important ones to include, which relate to the most widely used devices in the industry.

## Conclusion

For the first time, a standardised definition of minimalist shoes was developed through a consensus reached by an international panel of experts. In addition, the conception of the MI as a valid and reliable rating scale should be seen as a mutual effort to clarify the degree of minimalism of different shoe models using specific criteria. Given the strong panel of experts that participated to this consensus study, we recommend that the shoe industry use such standardised ratings to orientate the running community when selecting their running shoes among the wide range of available equipment. These findings will also help design and interpret future research pertaining to the effects of minimalist shoes on biomechanics and running-related injuries, and may help recreational runners and the medical community in decreasing injury rates due to inappropriate transition between running shoes.

## Additional files

Additional file 1:
**Minimalist Index rating scale.** (PDF 64 kb) Additional file 2:
**Minimalist Index instruction guide.** (PDF 524 kb)
