# Supplementary material for: A consensus definition and rating scale for minimalist shoes
Source: J Foot Ankle Res. 2015 Aug 19;8:42. doi: 10.1186/s13047-015-0094-5 (PMC4543477; doi:10.1186/s13047-015-0094-5)
Supplement: Additional file 1: — Minimalist Index rating scale. (PDF 64 kb) [file 13047_2015_94_MOESM1_ESM.pdf]

MINIMALIST INDEX

A) Weight: According to the scale, what is the weight of the shoe?

Weight = ☐ **grams**

5 = less than 125g
4 = from 125g to less than 175g
3 = from 175g to less than 225g
2 = from 225g to less than 275g
1 = from 275g to less than 325g
0 = 325g and more

Weight subscore =

B) Stack height: According to the caliper, what is the height of the shoe as measured in the middle of the heel?

Stack height = ☐ **mm**

5 = less than 8 mm
4 = from 8 mm to less than 14 mm
3 = from 14 mm to less than 20 mm
2 = from 20 mm to less than 26 mm
1 = from 26 mm to less than 32 mm
0 = 32 mm and more

Stack height subscore =

C) Heel to toe drop: According to the caliper, what is the height of the shoe under the metatarsal heads? Then, subtract this value from the stack height to obtain the heel to toe drop.

Stack height ☐ **mm**
- Height at forefoot ☐ **mm**
= Heel to toe drop ☐ **mm**

5 = less than 1 mm
4 = from 1mm to less than 4 mm
3 = from 4 mm to less than 7 mm
2 = from 7 mm to less than 10 mm
1 = from 10 mm to less than 13 mm
0 = 13 mm and more

Heel to toe drop subscore =

D) Stability and motion control technologies: Which of these technologies can you observe on the shoe?

- ☐ **Multi-density midsole**
- ☐ **Thermoplastic medial post**
- ☐ **Rigid heel counter**
- ☐ **Elevated medial insole under arch**
- ☐ **Supportive tensioned medial upper**
- ☐ **Medial flare**

5 = None
4 = 1 device
3 = 2 devices
2 = 3 devices
1 = 4 devices
0 = 5 or 6 devices

Stability and motion control technologies subscore =

E) Flexibility

Longitudinal flexibility: Using a pinch grip with thumb, index and middle fingers from both hands, apply a superiorly-directed force to the anterior and posterior parts of the shoe. How would you rate longitudinal flexibility?

☐

2.5 = Minimal resistance to longitudinal bending (the shoe can be rolled on itself more than 360 degrees)
2.0 = Slight resistance to longitudinal bending (anterior tip of shoe sole reaches posterior tip of shoe sole in a maximal bending of 360 degrees)
1.5 = Moderate resistance to longitudinal bending (anterior tip of shoe sole doesn't reach posterior tip of shoe sole, but anterior and posterior parts of the shoe can form an angle of at least 90 degrees)
1.0 = High resistance to longitudinal bending (anterior and posterior parts of the shoe can form an angle between 45 and 90 degrees)
0.5 = Very high resistance to longitudinal bending (longitudinal deformation is possible, but anterior and posterior parts of the shoe form a maximum angle of 45 degrees)
0 = Extreme resistance to longitudinal bending (longitudinal forces don't significantly change the orientation of the anterior part of the shoe relative to the posterior part)

Torsional flexibility: Using a pinch grip with thumb, index and middle fingers from both hands, apply a medially-directed torsional force (pronation) to the anterior part of the shoe. How would you rate torsional flexibility?

☐

2.5 = Minimal resistance to torsion (anterior part of the shoe is turned 360 degrees; anterior outsole faces inferiorly after a complete twist while posterior outsole faces inferiorly)
2.0 = Slight resistance to torsion (anterior part of the shoe is turned at least 180 degrees but less than 360 degrees; anterior outsole faces at least superiorly while posterior outsole faces inferiorly)
1.5 = Moderate resistance to torsion (anterior part of the shoe is turned more than 90 degrees but less than 180 degrees; anterior outsole faces at least laterally while posterior outsole faces inferiorly)
1.0 = High resistance to torsion (anterior part of the shoe is turned more than 45 degrees but less than 90 degrees; anterior outsole can't face laterally while posterior outsole faces inferiorly)
0.5 = Very high resistance to torsion (torsional deformation is possible, but anterior part of the shoe reaches less than 45 degrees)
0 = Extreme resistance to torsion (torsional forces don't significantly change the orientation of the anterior part of the shoe relative to the posterior part)

Flexibility subscore (sum of torsional and longitudinal) =

Sum of subscores = ________________ x 4 = ______________% (Total MI score)
