# Supplementary material for: A consensus definition and rating scale for minimalist shoes
Source: J Foot Ankle Res. 2015 Aug 19;8:42. doi: 10.1186/s13047-015-0094-5 (PMC4543477; doi:10.1186/s13047-015-0094-5)

UNIVERSITÉ LAVAL

Instructions on how to rate running shoes using the

MINIMALIST INDEX

The Minimalist Index was developed following an expert consensus led by

Jean-Francois ESCULIER, Blaise DUBOIS, Jean-Sébastien ROY & Clermont E. DIONNE

June 2014

A) WEIGHT

Place the shoe on the scale. According to the scale, what is the weight of the shoe (in grams)?

5/5 = less than 125g

4/5 = from 125g to less than 175g

3/5 = from 175g to less than 225g

2/5 = from 225g to less than 275g

1/5 = from 275g to less than 325g

0/5 = 325g and more

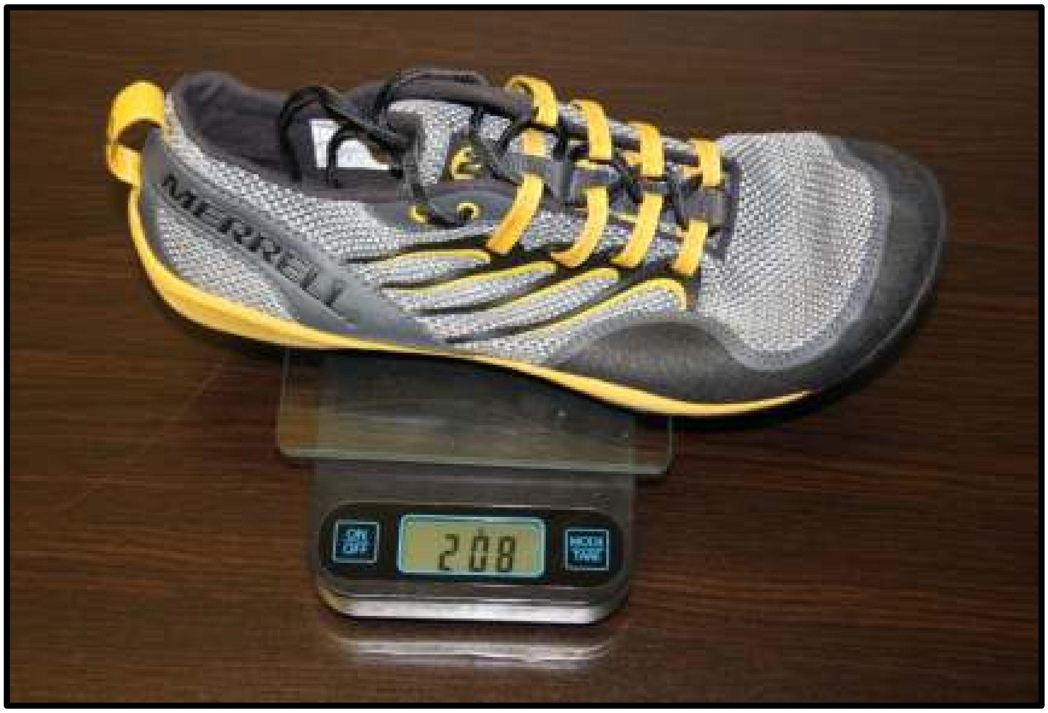

Fig A-1. Weighting of the shoe using a digital scale.

B) STACK HEIGHT

Using a digital caliper, measure the height of the shoe at the heel (including insole, midsole and outsole). The caliper must be placed at the middle of the heel when looking at the shoe from the rear end, and in the middle of the heel when looking at the shoe from the side.

Caution must be taken when placing the caliper on the outsole, as the most external (thickest) point of the shoe has to be considered.

5/5 = less than 8 mm

4/5 = from 8 mm to less than 14 mm

3/5 = from 14 mm to less than 20 mm

2/5 = from 20 mm to less than 26 mm

1/5 = from 26 mm to less than 32 mm

0/5 = 32 mm and more

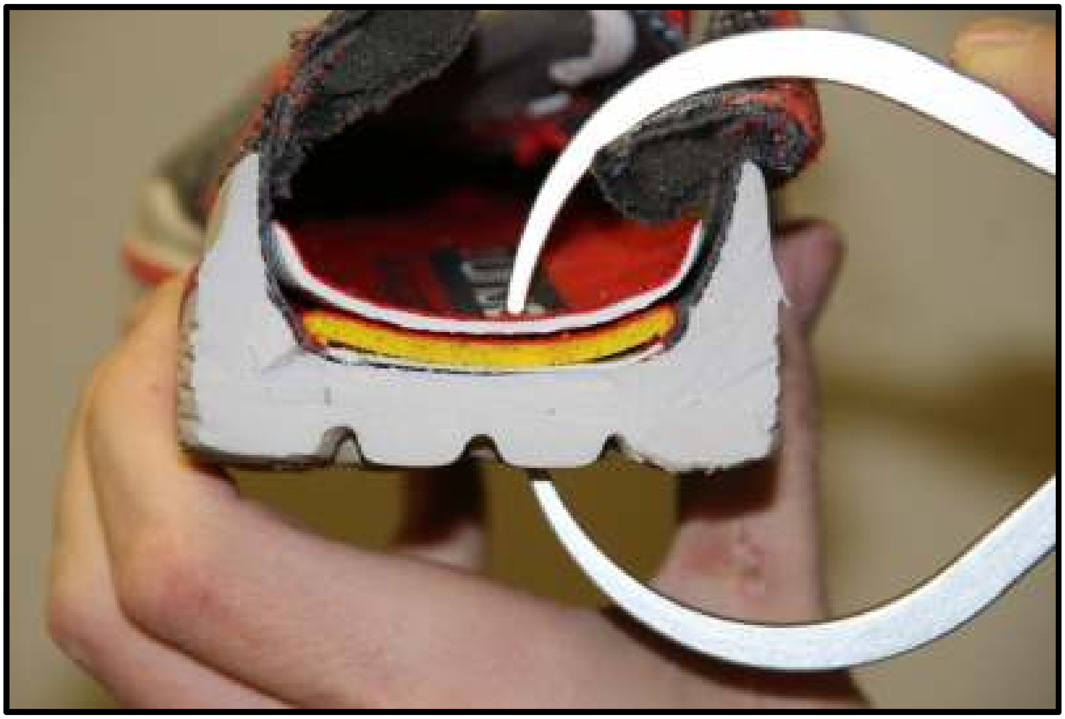

Fig B-1. The caliper must be placed at the middle of the shoe when looking from the rear end.

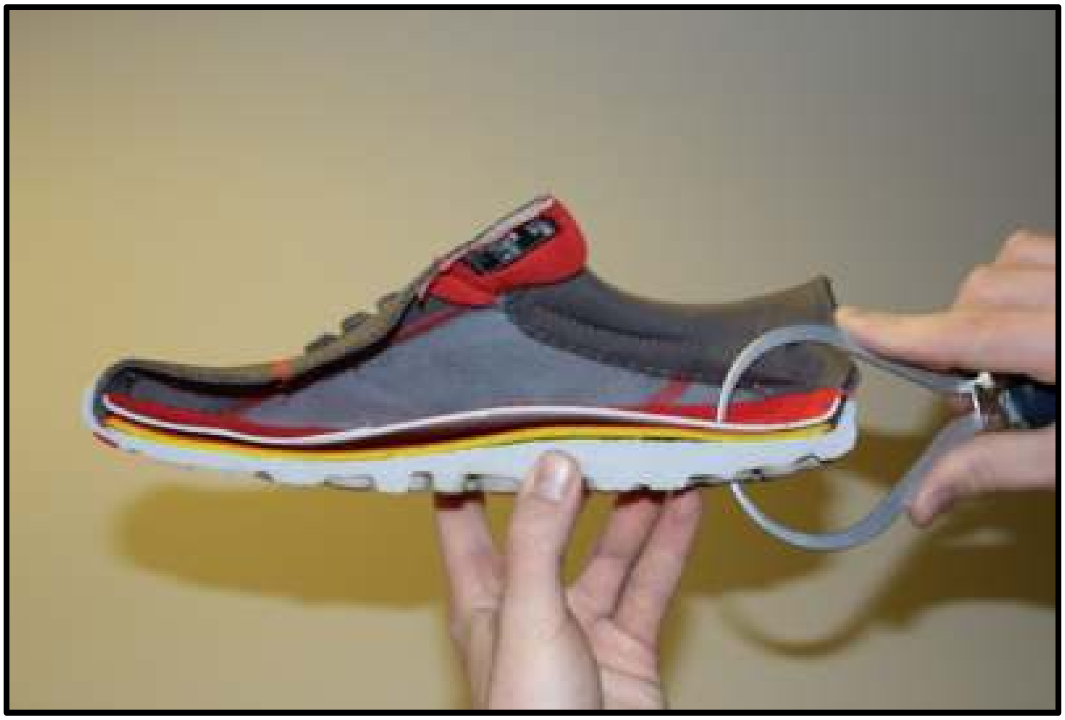

Fig B-2. The caliper must be placed at the middle of the heel when looking from the side

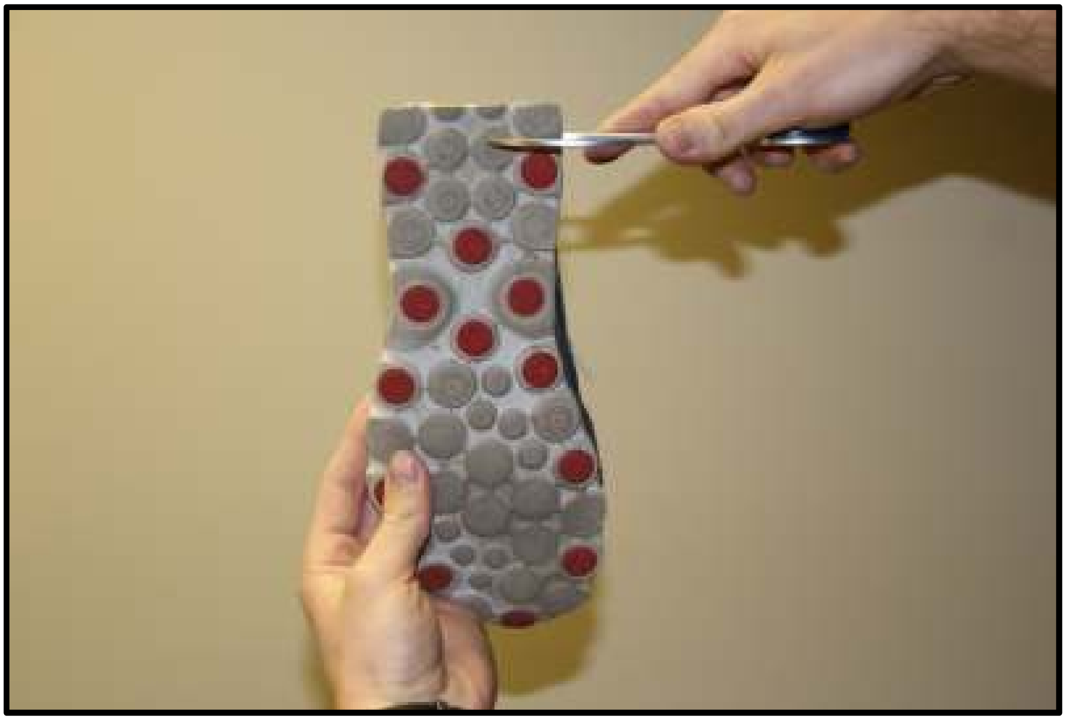

Fig B-3. The caliper must be placed at the most external (thickest) point of the shoe.

C) HEEL TO TOE DROP

Using a digital caliper, measure the height of the shoe at the metatarsal heads (including insole, midsole and outsole). The caliper must be placed at the middle of the shoe when looking at the shoe from the top. Caution must be taken when placing the caliper on the outsole, as the most external (thickest) point of the shoe has to be considered.

Now, substract the height at the metatarsal heads from the stack height to obtain the heel to toe drop.

5/5 = less than 1 mm

4/5 = from 1 mm to less than 4 mm

3/5 = from 4 mm to less than 7 mm

2/5 = from 7 mm to less than 10 mm

1/5 = from 10 mm to less than 13 mm

0/5 = 13 mm and more

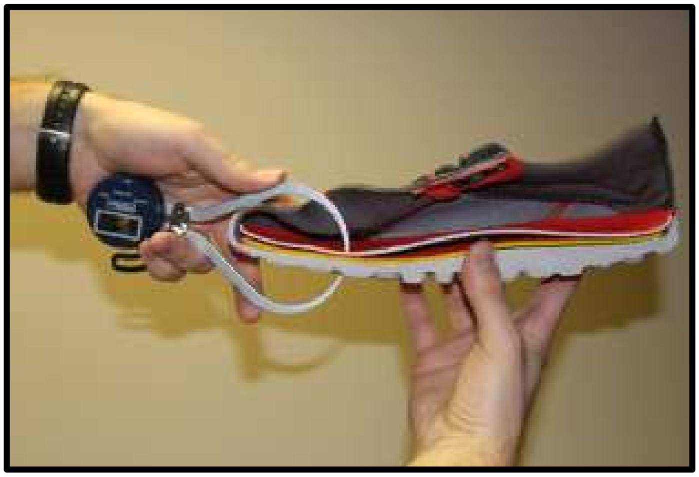

Fig C-1. The caliper must be placed under the metatarsal heads

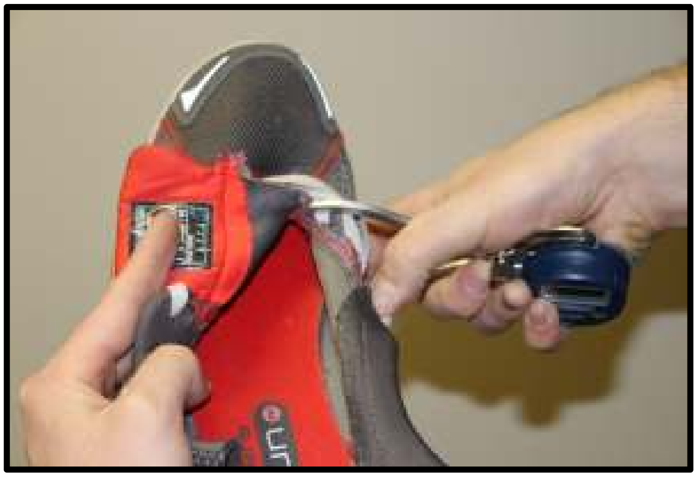

Fig C-2. The caliper must be placed at the middle of the shoe when looking from the top

D) MOTION CONTROL AND STABILITY TECHNOLOGIES

Which of these technologies (see pictures) can you observe on the shoe?

- Multi-density midsole
- Thermoplastic medial post
- Rigid heel counter
- Elevated medial insole under arch
- Supportive tensioned medial upper
- Medial flare

5/5 = None

4/5 = 1 device

3/5 = 2 devices

2/5 = 3 devices

1/5 = 4 devices

0/5 = 5 or 6 devices

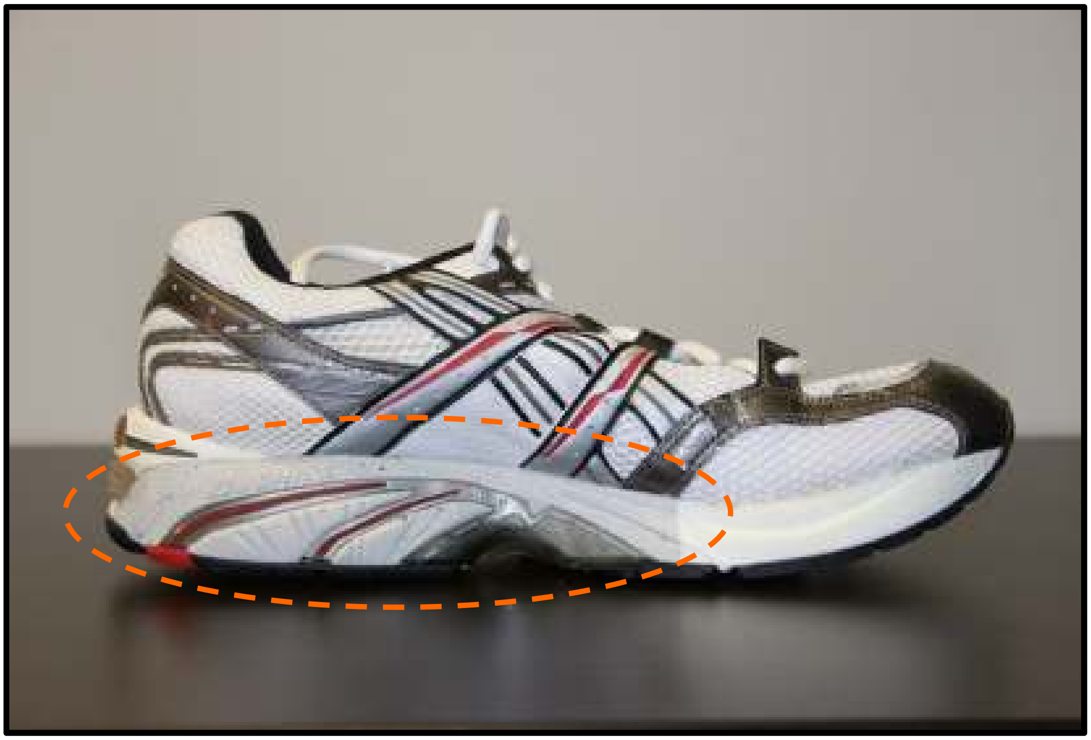

Fig D-1. Multi-density midsole: Typically, a different color is used to emphasize this feature.

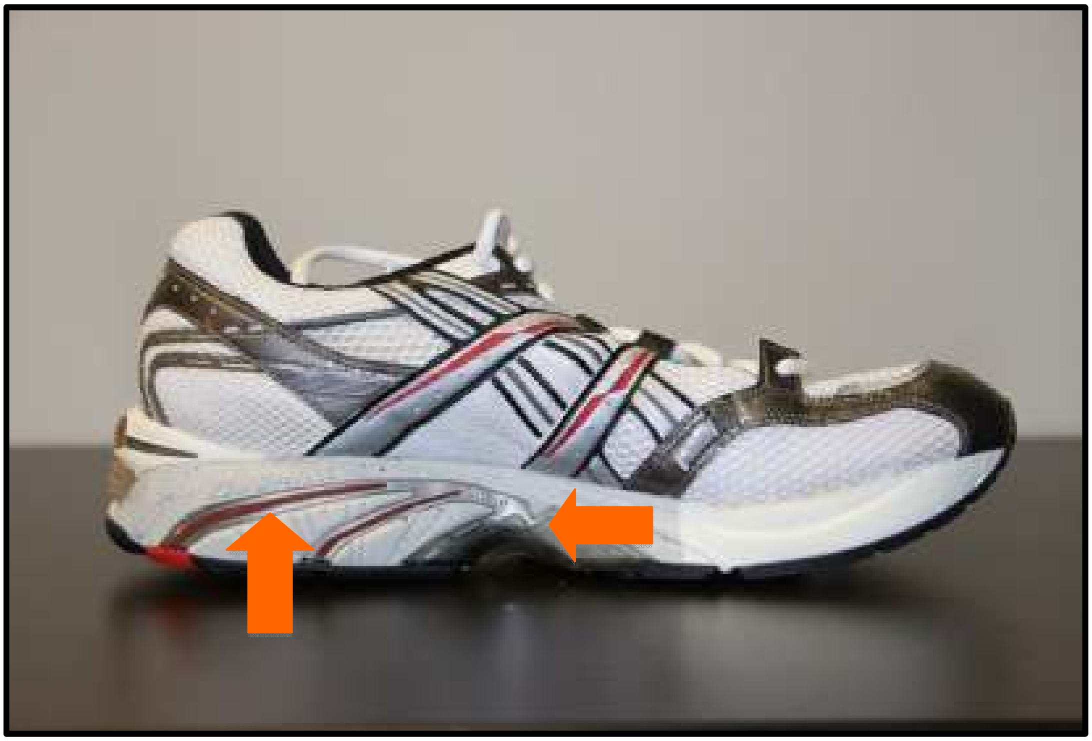

Fig D-2. Thermoplastic medial post. Plastic is used to reinforce the medial portion of midsole.

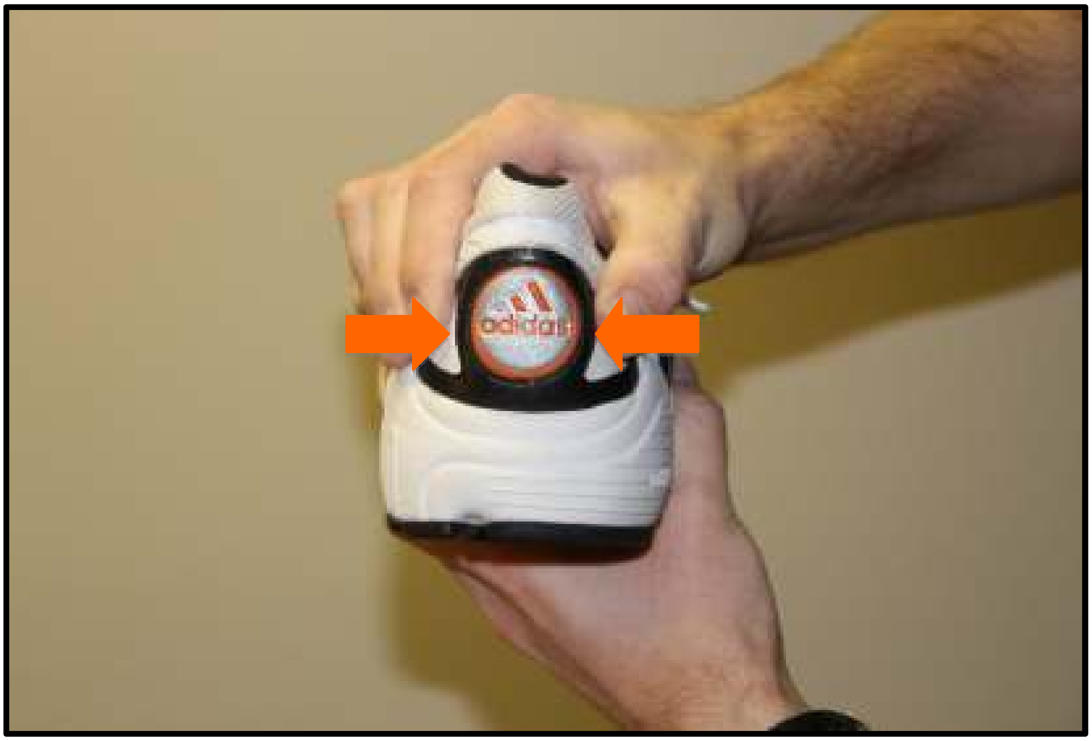

Fig D-3. Rigid heel counter.

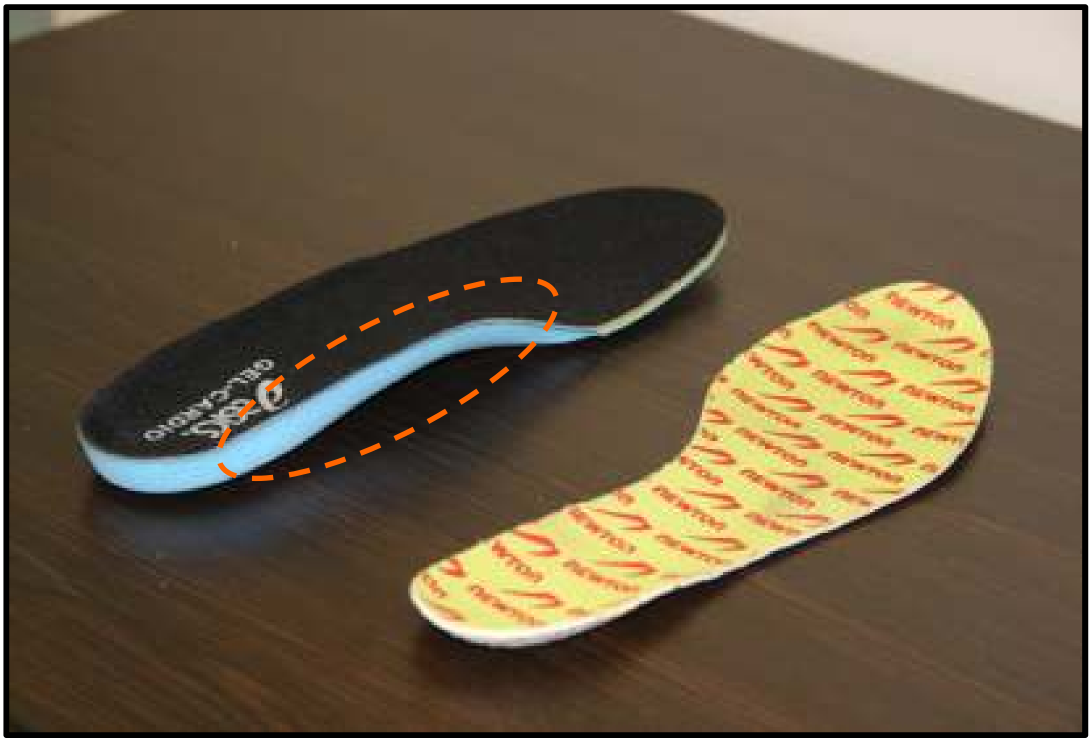

Fig D-4. Elevated medial insole under arch (left), compared with a flat insole (right).

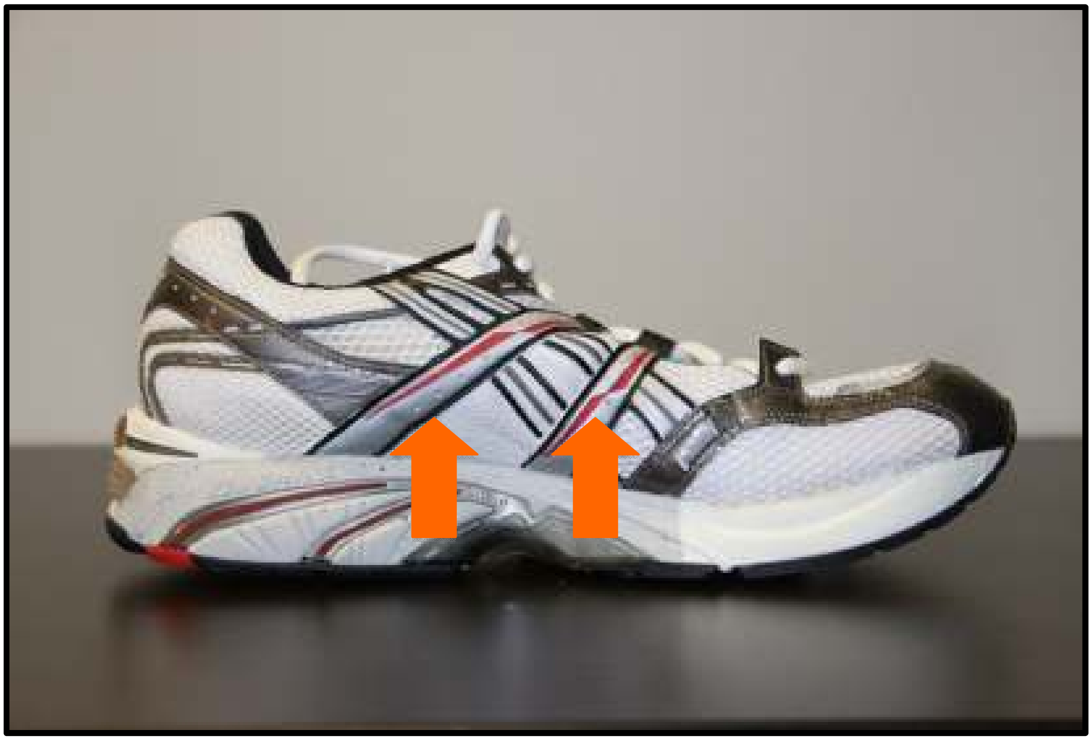

Fig D-5. Supportive tensioned medial upper. Material is used to reinforce medial upper in order to limit medial foot movement.

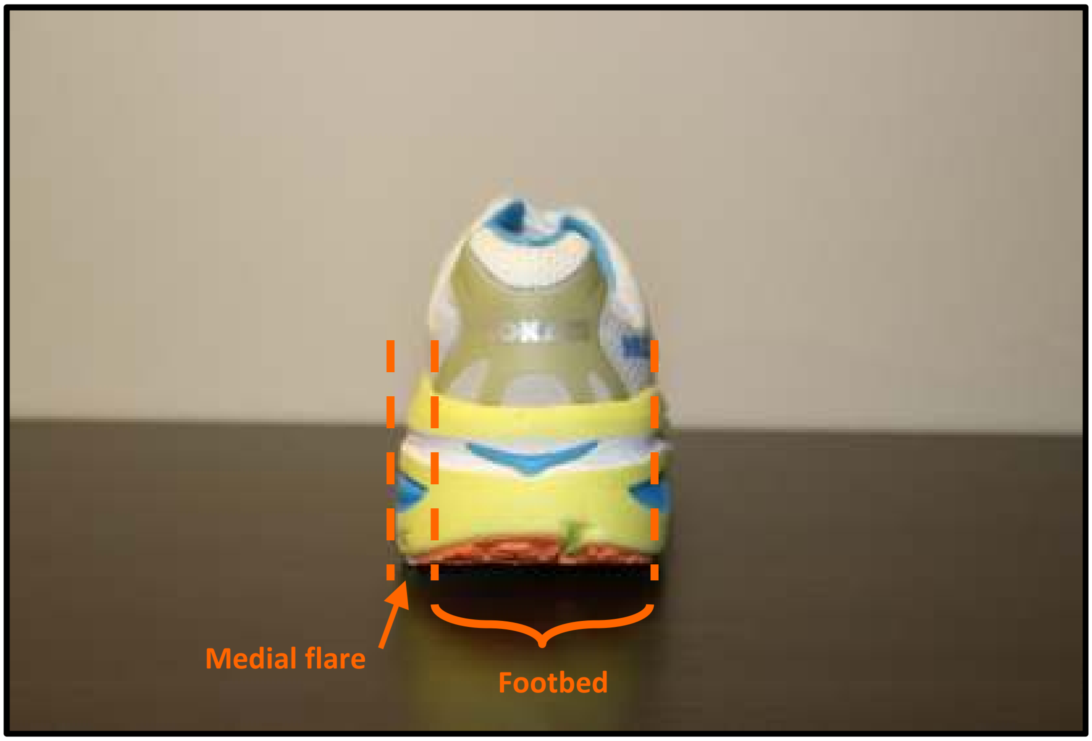

Fig D-6. Medial flare. Medial tip of midsole extends beyond footbed.

E) FLEXIBILITY

Longitudinal flexibility

Using a pinch grip with thumb, index and middle fingers from both hands, apply a superiorly-directed force to the anterior and posterior parts of the shoe. See images below to determine appropriate rating.

2.5/2.5

Minimal resistance to longitudinal bending (the shoe can be rolled on itself more than 360 degrees)

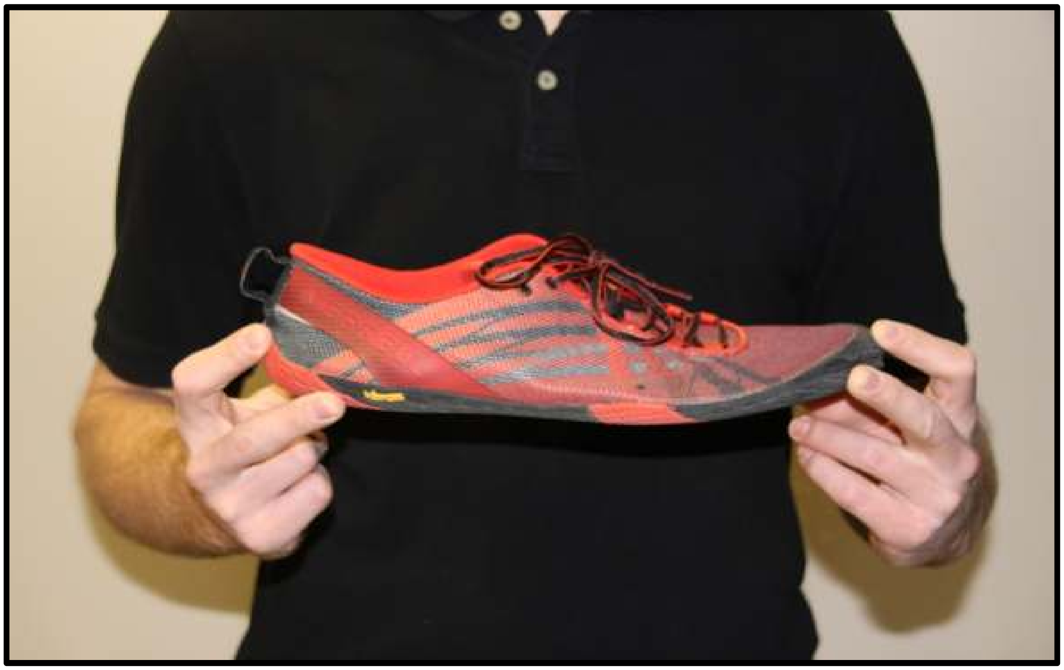

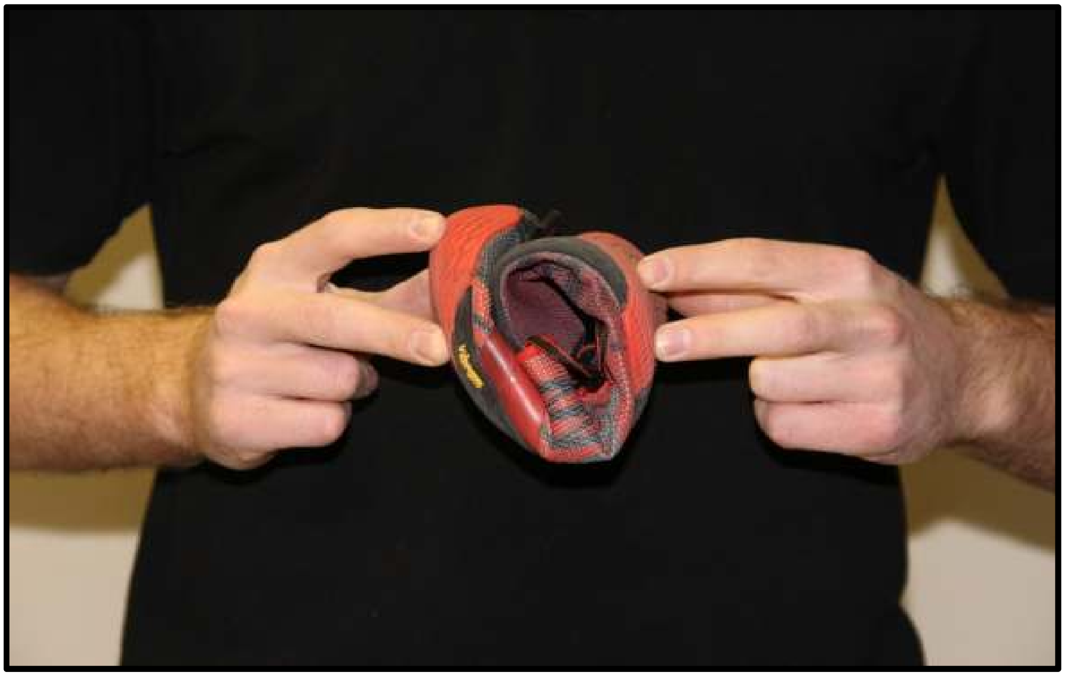

Longitudinal flexibility

2.0/2.5

Slight resistance to longitudinal bending (anterior tip of shoe sole reaches posterior tip of shoe sole in a maximal bending of 360 degrees)

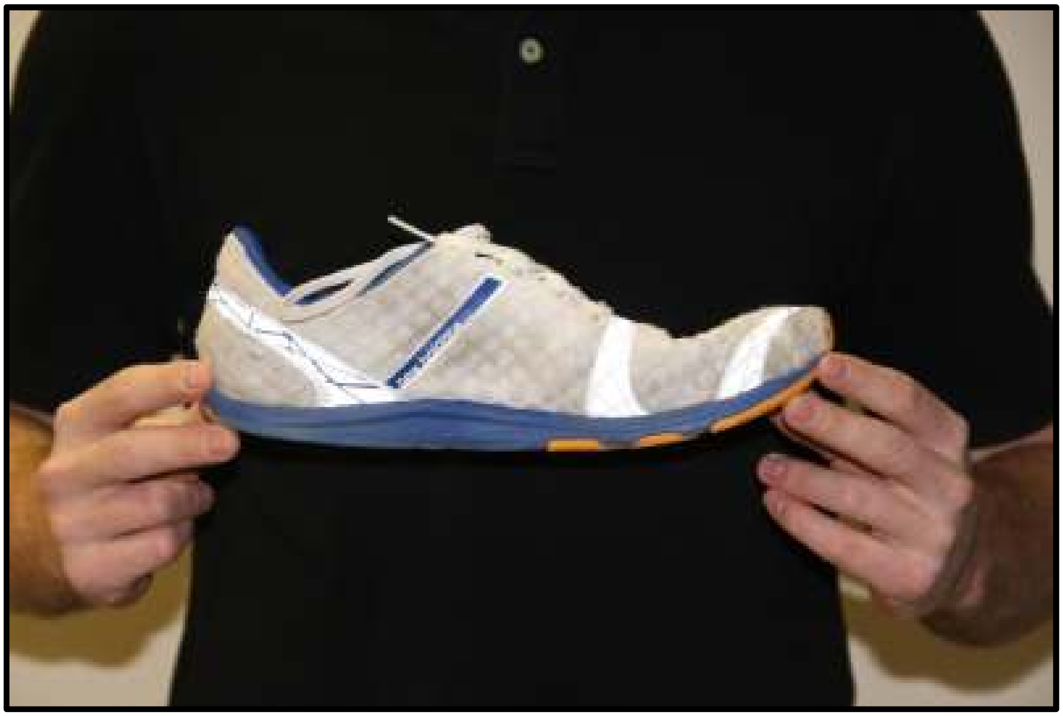

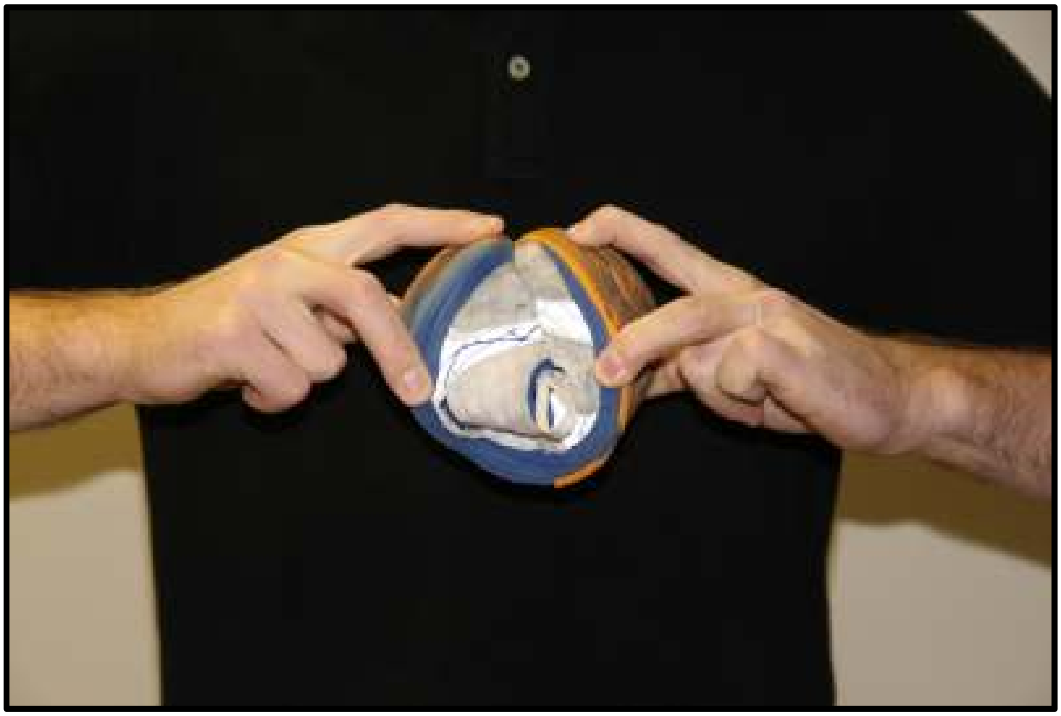

Longitudinal flexibility

1.5/2.5

Moderate resistance to longitudinal bending (anterior tip of shoe sole doesn't reach posterior tip of shoe sole, but anterior and posterior parts of the shoe can form an angle of at least 90 degrees)

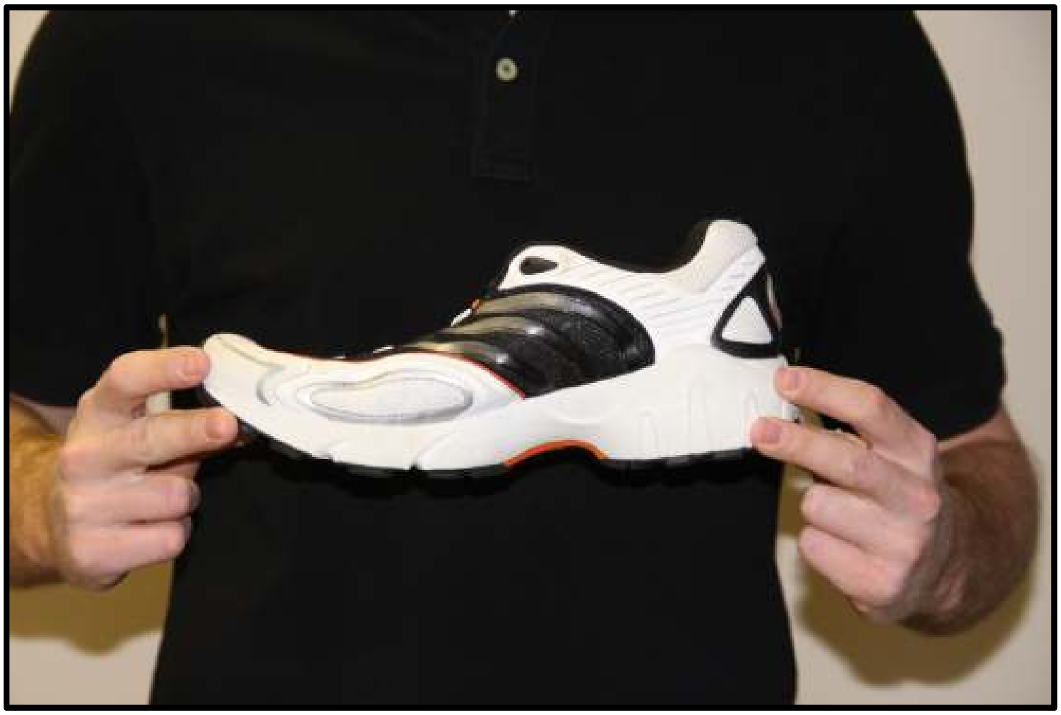

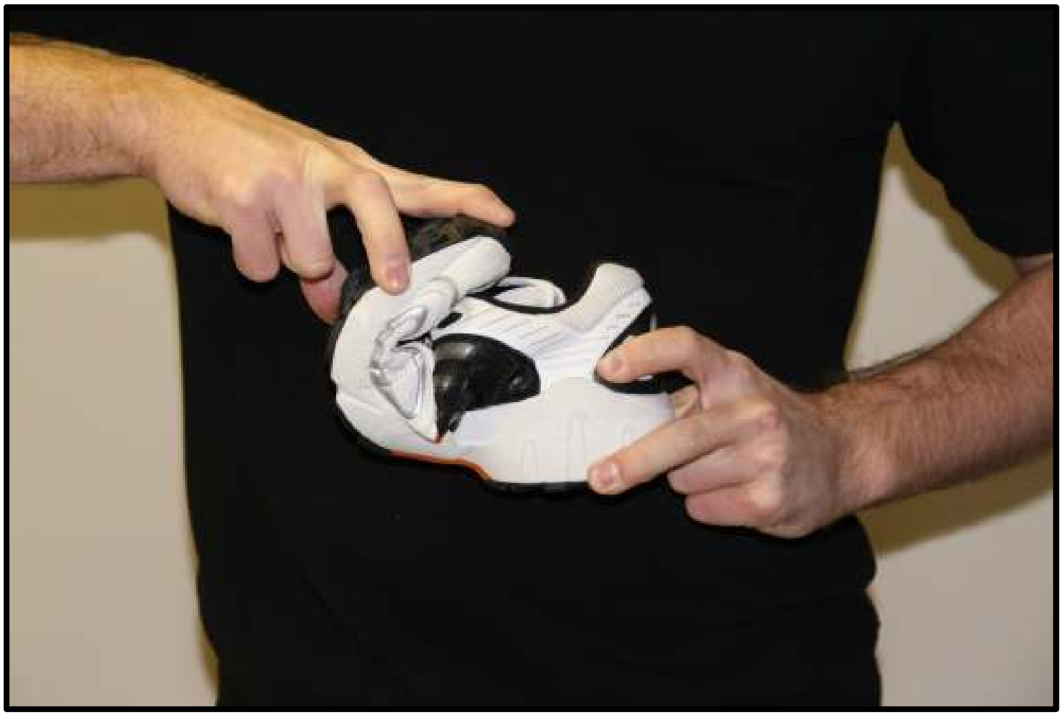

Longitudinal flexibility

1.0/2.5

High resistance to longitudinal bending (anterior and posterior parts of the shoe can form an angle between 45 and 90 degrees)

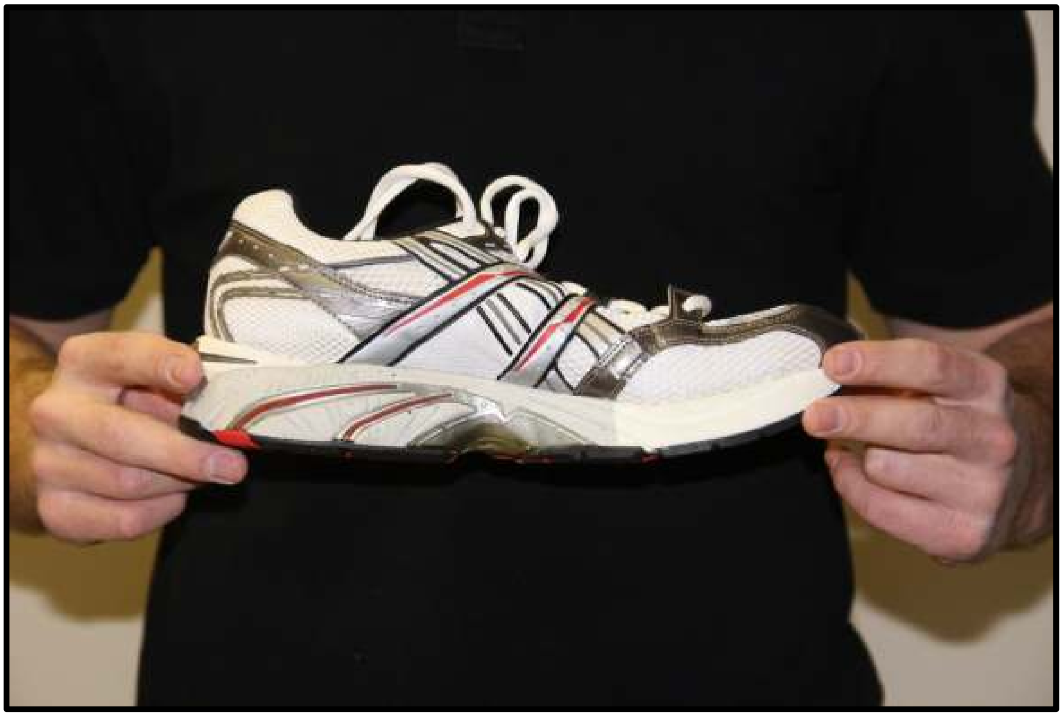

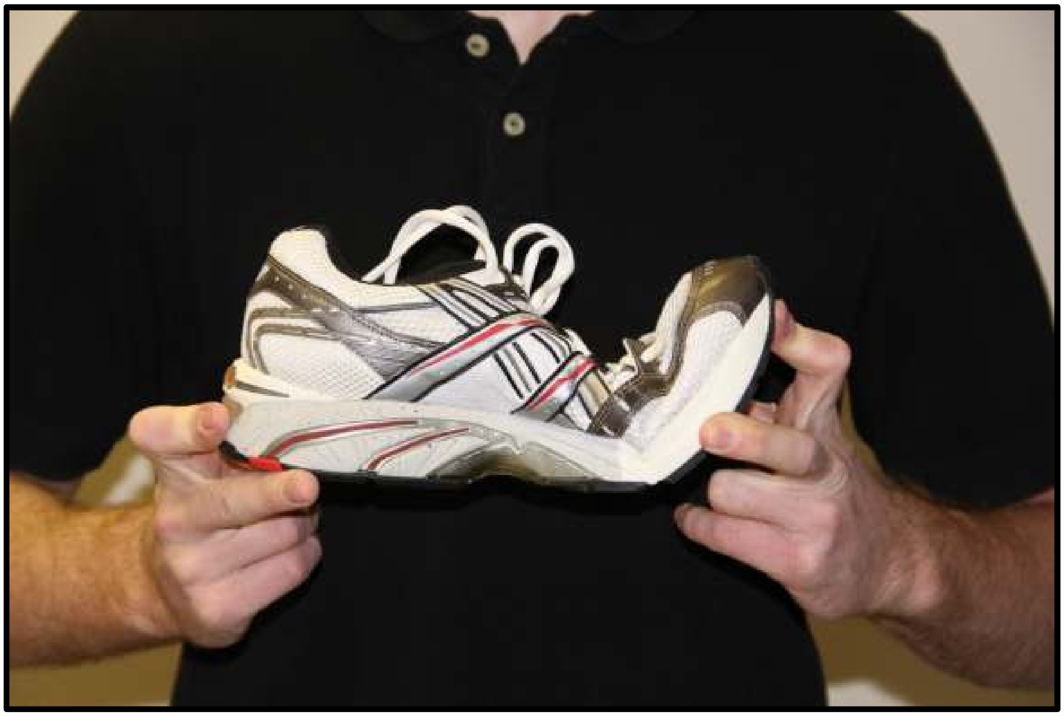

Longitudinal flexibility

0.5/2.5

Very high resistance to longitudinal bending (longitudinal deformation is possible, but anterior and posterior parts of the shoe form a maximum angle of 45 degrees)

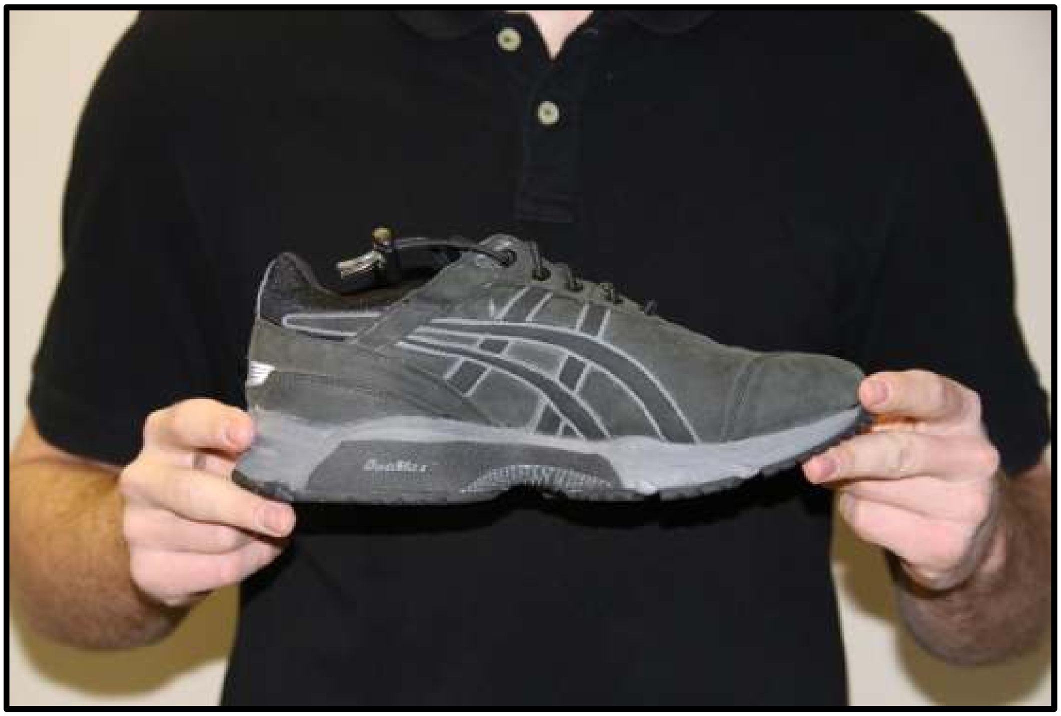

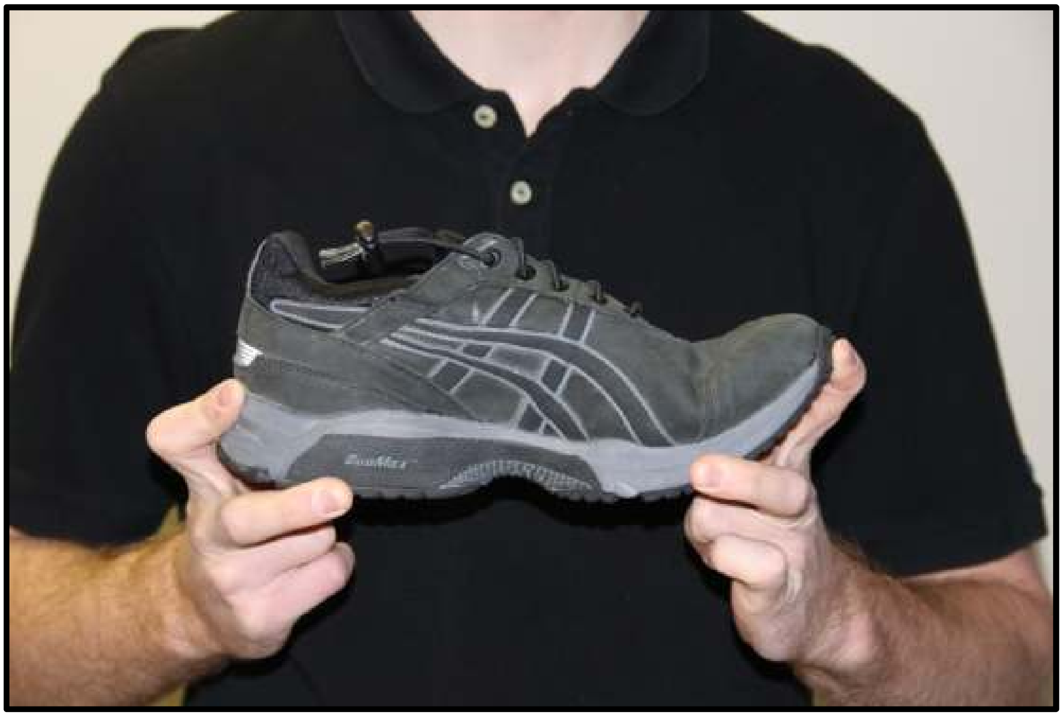

Longitudinal flexibility

0/2.5

Extreme resistance to longitudinal bending (longitudinal forces don't significantly change the orientation of the anterior part of the shoe relative to the posterior part)

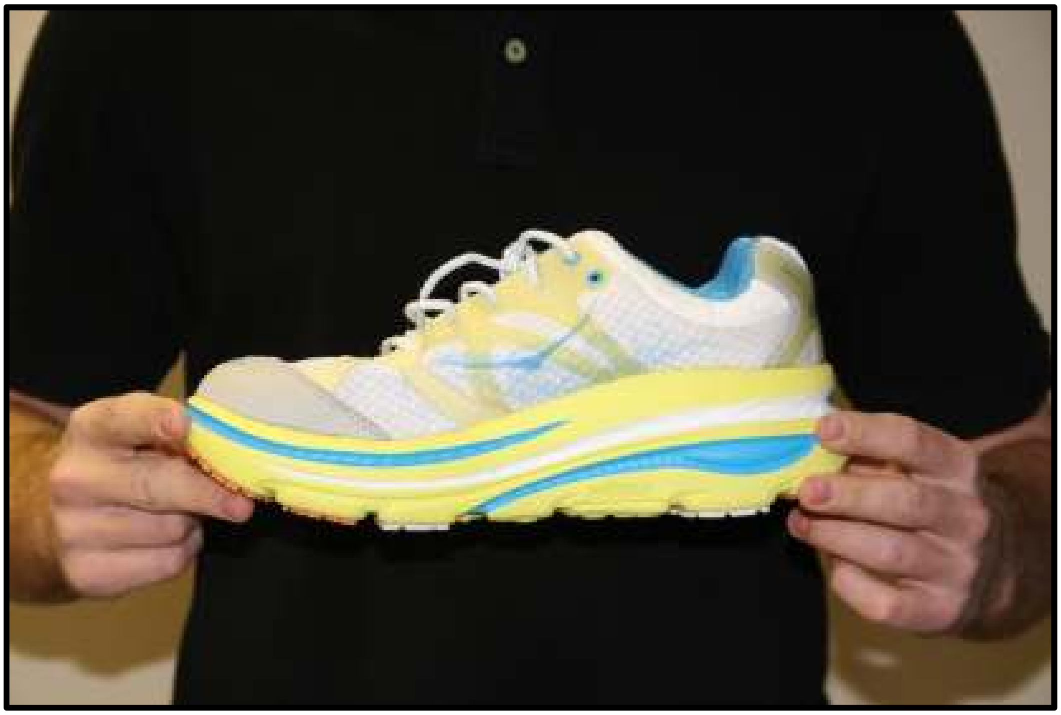

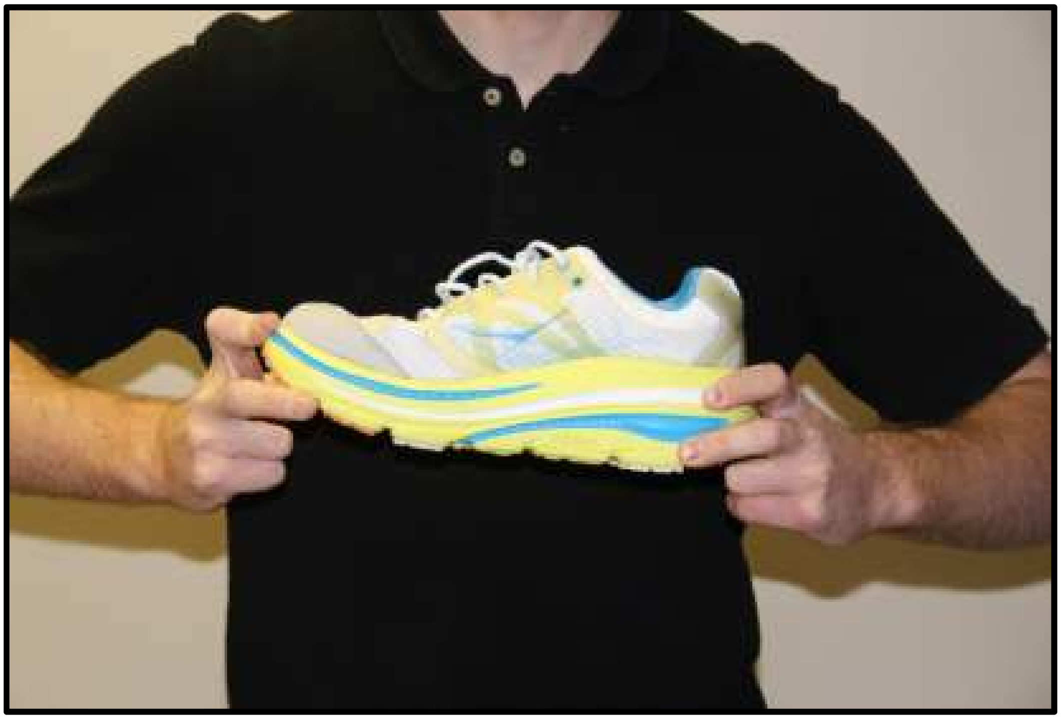

Torsional flexibility

Using a pinch grip with thumb, index and middle fingers from both hands, apply a medially-directed torsional force (pronation) to the anterior part of the shoe. See images below to determine appropriate rating.

2.5/2.5

Minimal resistance to torsion (anterior part of the shoe is turned 360 degrees; anterior outsole faces inferiorly after a complete twist while posterior outsole faces inferiorly)

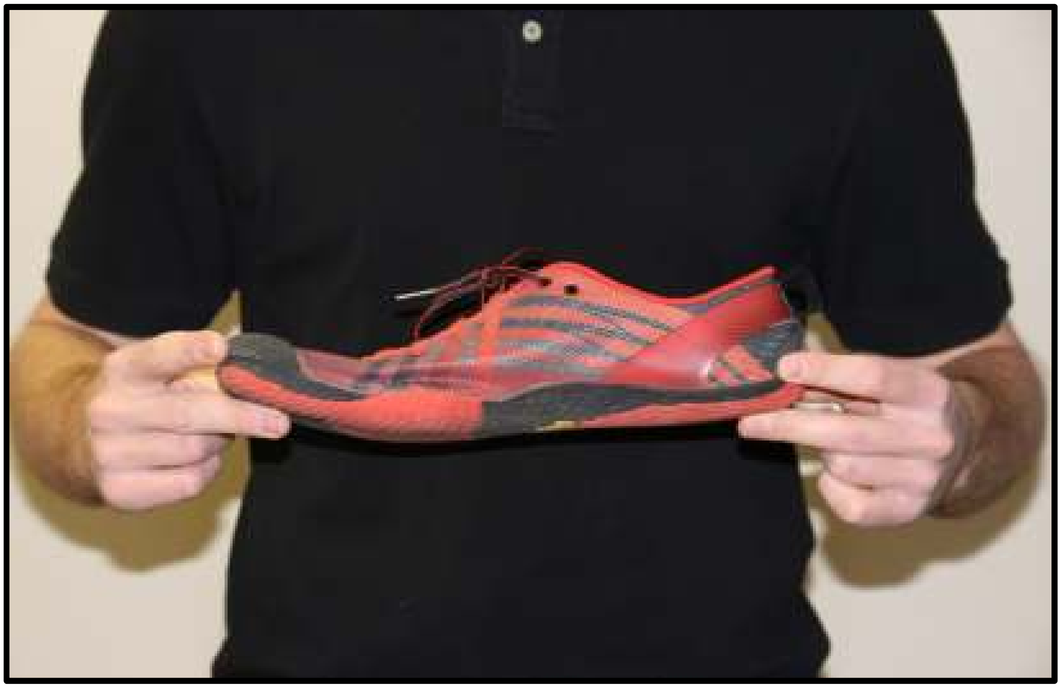

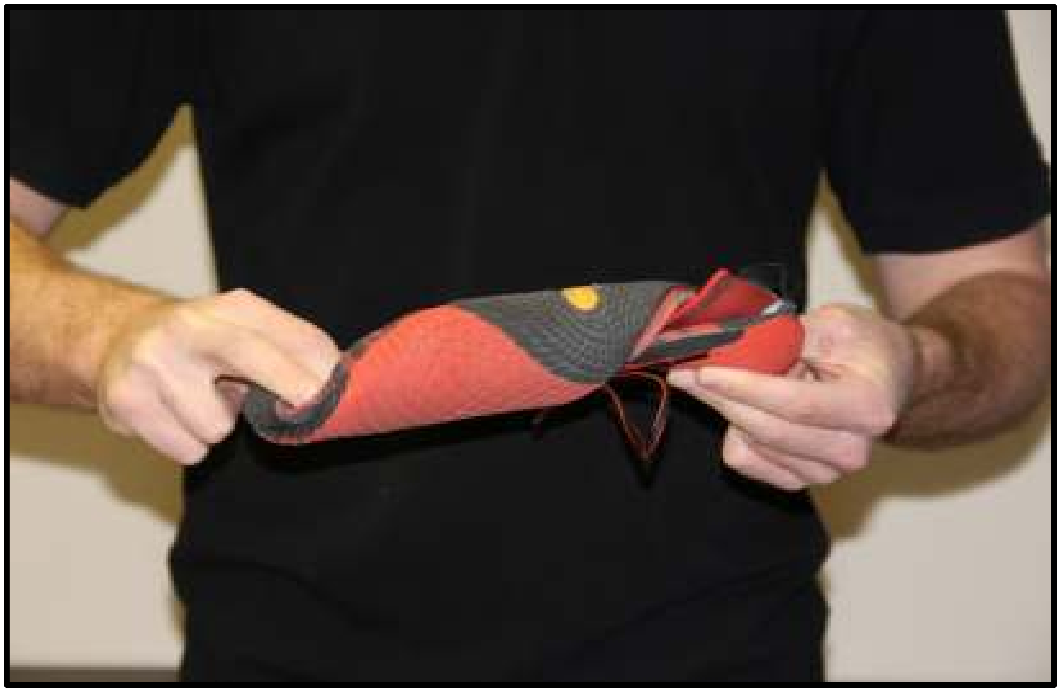

Torsional flexibility

2.0/2.5

Slight resistance to torsion (anterior part of the shoe is turned at least 180 degrees but less than 360 degrees; anterior outsole faces at least superiorly while posterior outsole faces inferiorly)

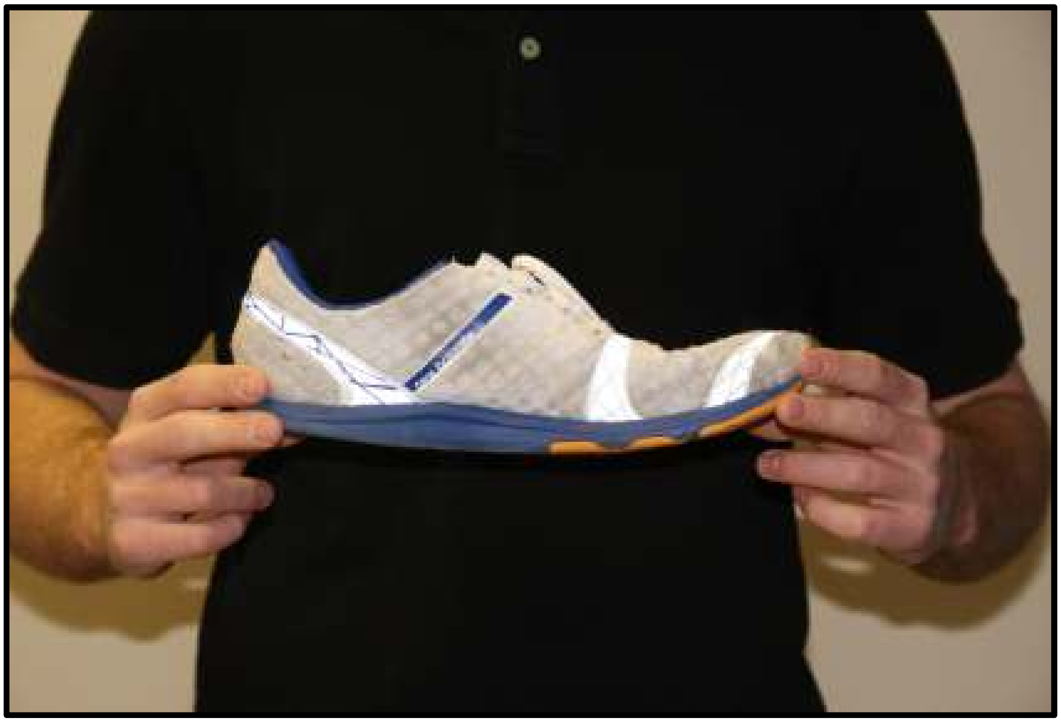

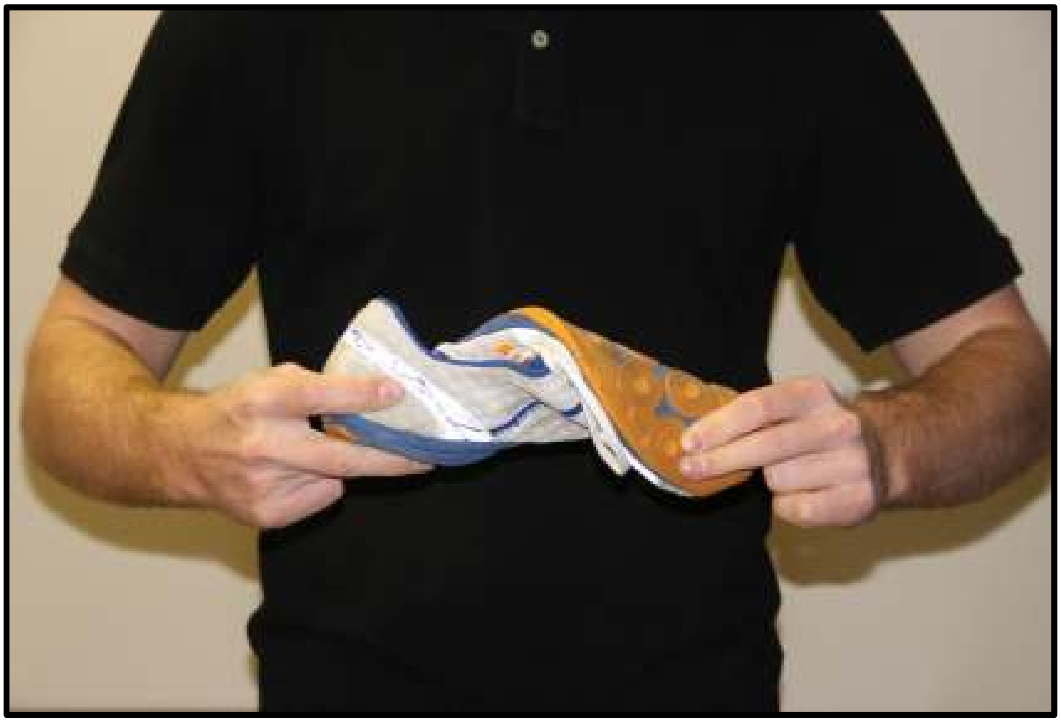

Torsional flexibility

1.5/2.5

Moderate resistance to torsion (anterior part of the shoe is turned more than 90 degrees but less than 180 degrees; anterior outsole faces at least laterally while posterior outsole faces inferiorly)

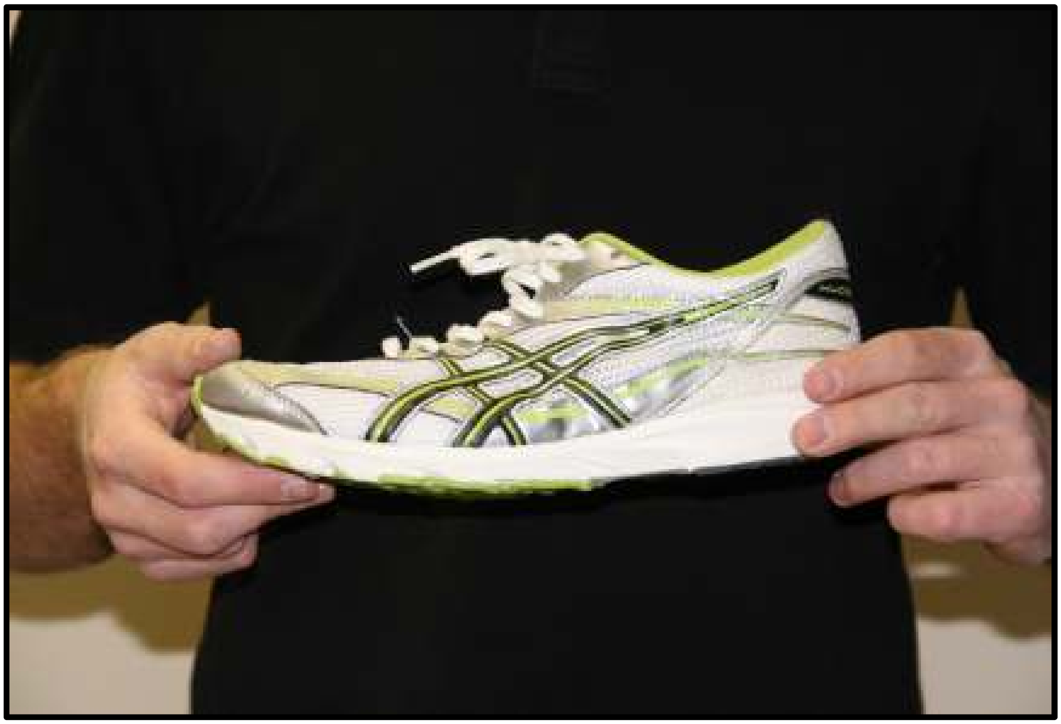

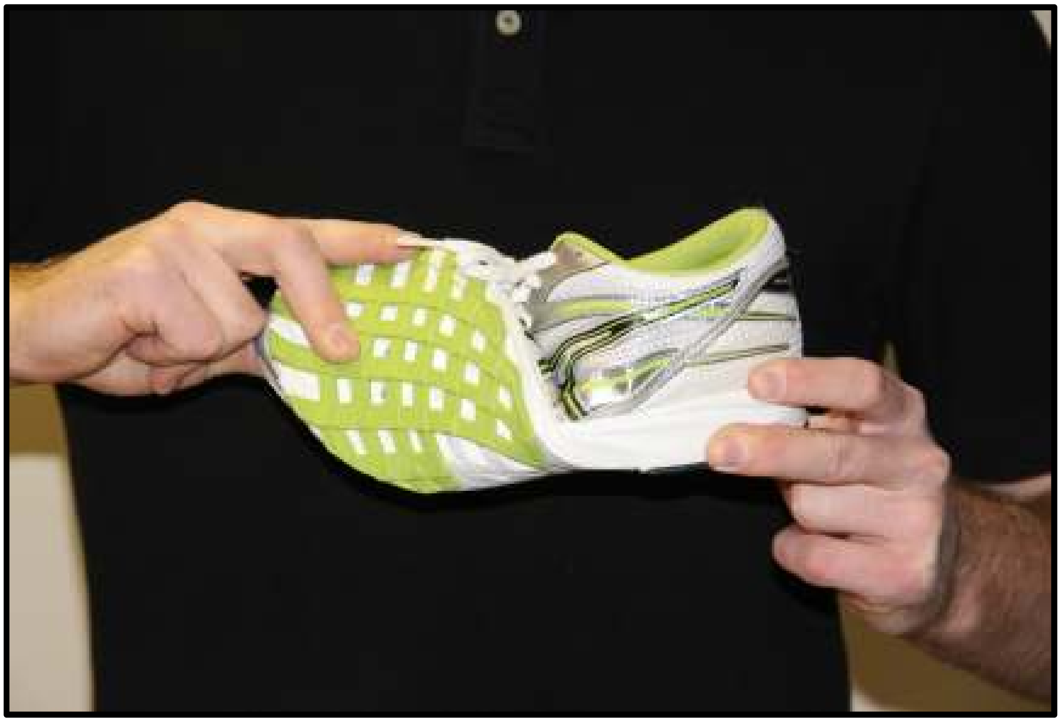

Torsional flexibility

1.0/2.5

High resistance to torsion (anterior part of the shoe is turned more than 45 degrees but less than 90 degrees; anterior outsole can't face laterally while posterior outsole faces inferiorly)

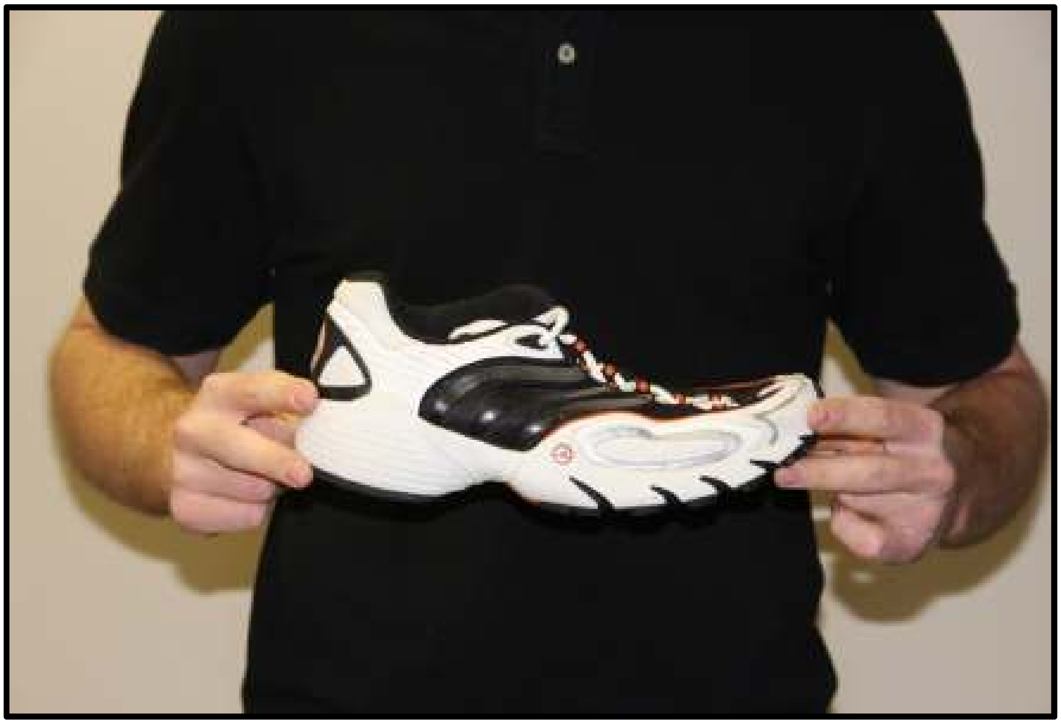

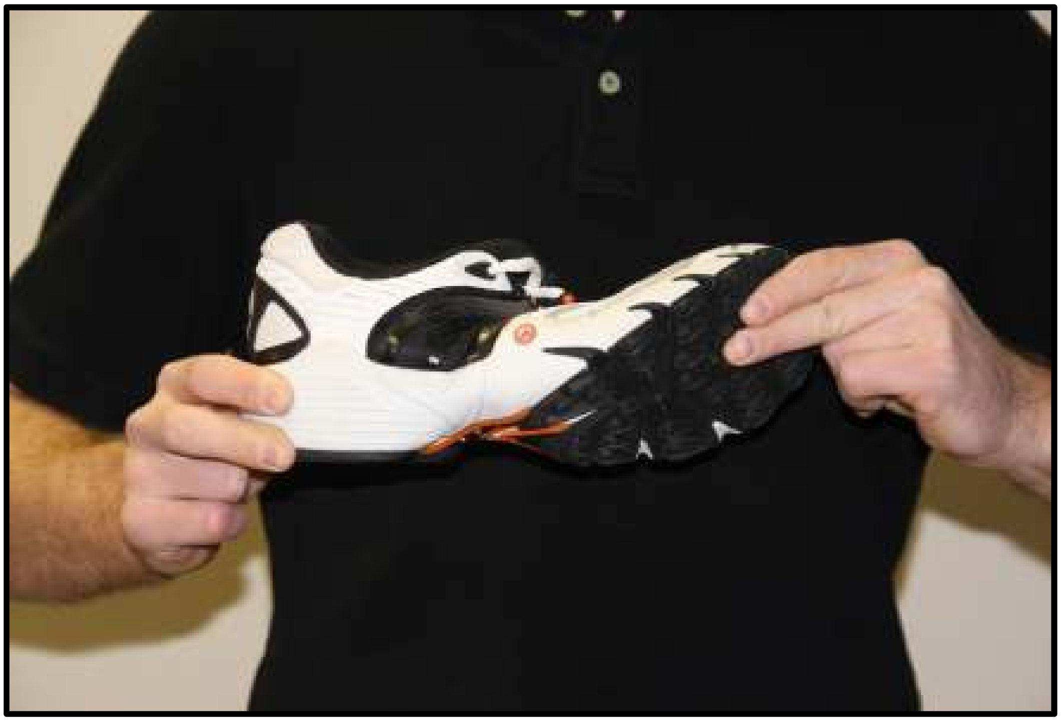

Torsional flexibility

0.5/2.5

Very high resistance to torsion (torsional deformation is possible, but anterior part of the shoe reaches less than 45 degrees

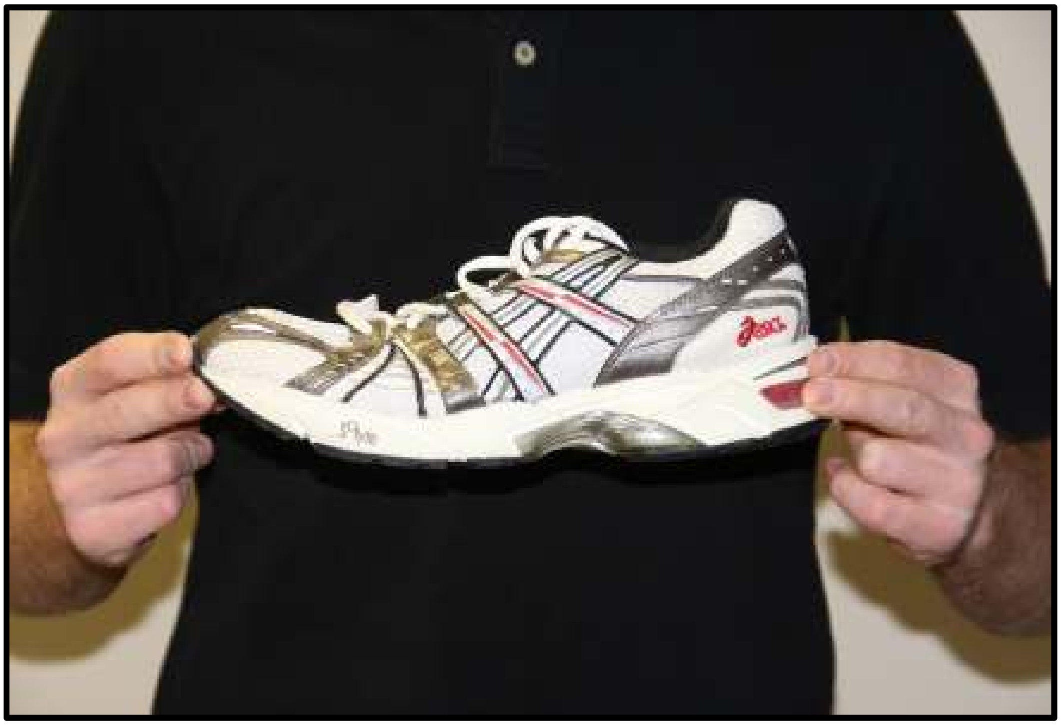

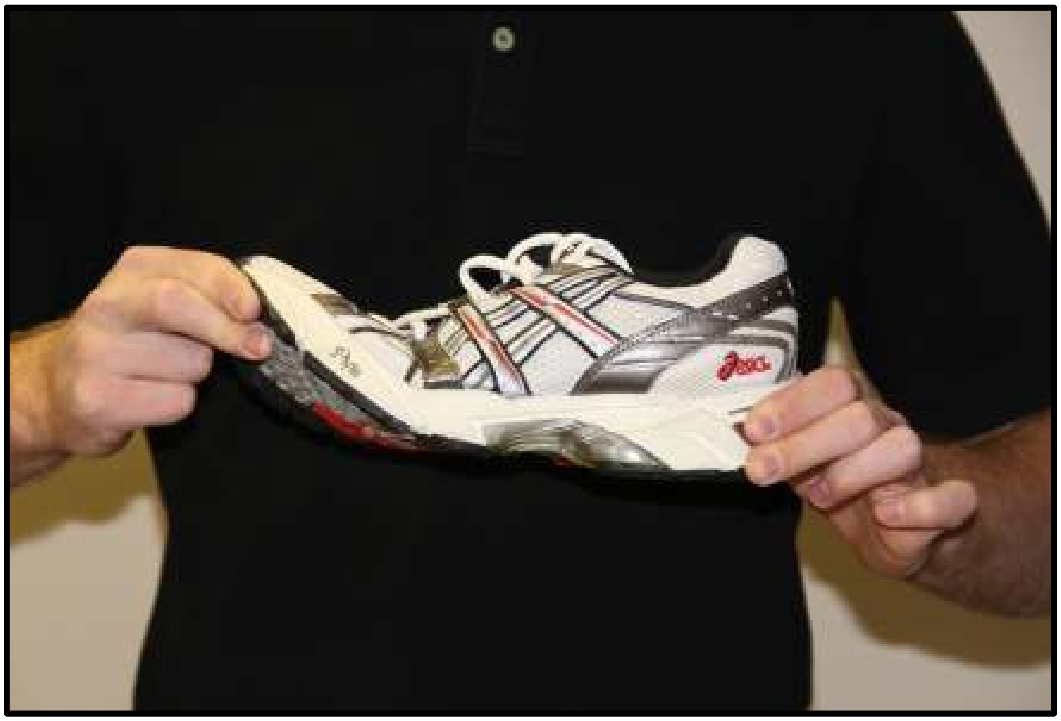

Torsional flexibility

0/2.5

Extreme resistance to torsion (torsional forces don't significantly change the orientation of the anterior part of the shoe relative to the posterior part)

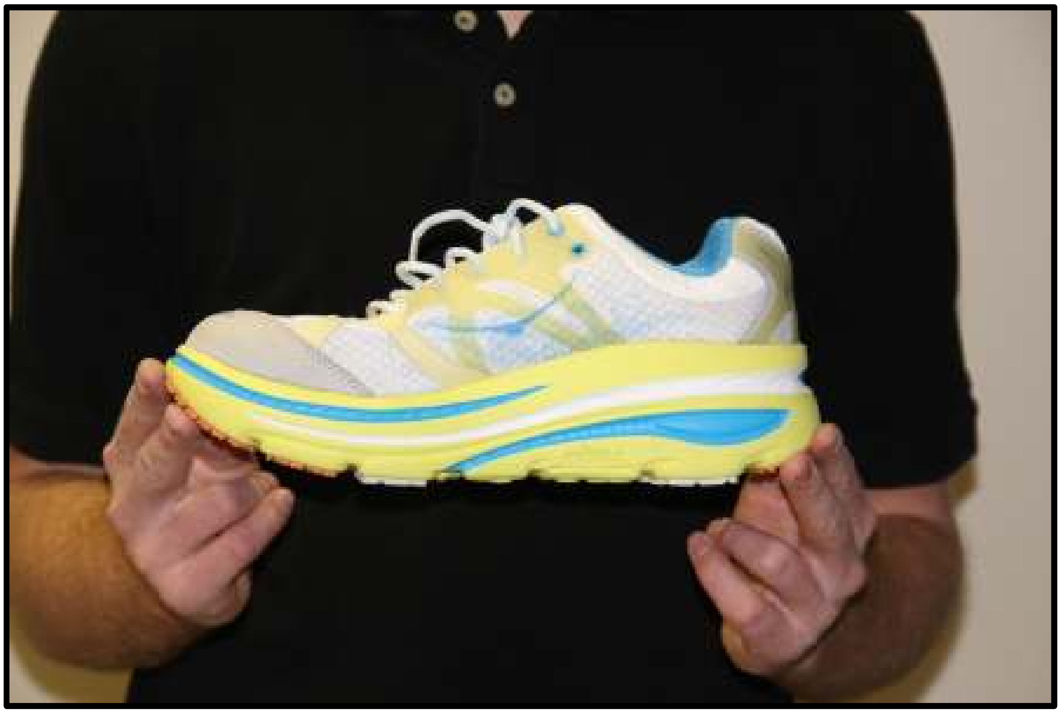

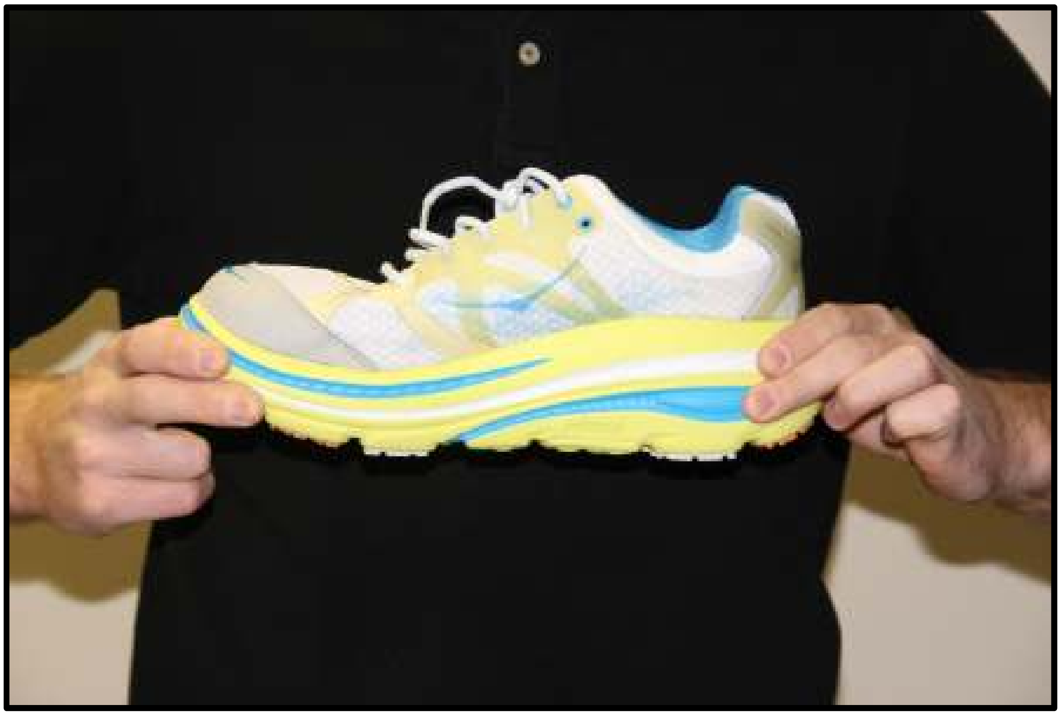

Supplement: Additional file 2: — Minimalist Index instruction guide. (PDF 524 kb) [file 13047_2015_94_MOESM2_ESM.pdf]
